# Haploid Mutation Mapping Identifies a Homoeologous Non‐Reciprocal Translocation Linked to Reduced Fibre and Enhanced Protein in *Brassica napus*

**DOI:** 10.1111/pbi.70535

**Published:** 2026-01-29

**Authors:** Morgan W. Kirzinger, Sarika Saini, Andrea T. Todd, Ushan Alahakoon, Kevin C. Koh, Justin B. Nichol, HaiYing Yuan, Kevin Fengler, Victor Llaca, Dustin Cram, Sampath Perumal, Wali Soomro, Magda Konopka, Tancey Melchkart, Venkat Bandi, Yasmina Bekkaoui, Yifang Tan, Chad Matsalla, Andrew G. Sharpe, Carl Gutwin, Fred Thoonen, Igor Falak, Chad Koscielny, Stuart Gardner, Isobel A. P. Parkin, Marcus A. Samuel, Alison M. R. Ferrie, Dave Charne, Daoquan Xiang, Jetty S. S. AmmiRaju, Sateesh Kagale

**Affiliations:** ^1^ Aquatic and Crop Resource Development National Research Council Canada Saskatoon Saskatchewan Canada; ^2^ Corteva Agriscience Caledon Ontario Canada; ^3^ Corteva Agriscience Saskatoon Saskatchewan Canada; ^4^ Global Institute for Food Security University of Saskatchewan Saskatoon Saskatchewan Canada; ^5^ Department of Biological Sciences University of Calgary Calgary Alberta Canada; ^6^ Corteva Agriscience Johnston Iowa USA; ^7^ Department of Computer Science University of Saskatchewan Saskatoon Saskatchewan Canada; ^8^ Corteva Agriscience Carmen Manitoba Canada; ^9^ Agriculture and Agri‐Food Canada Saskatoon Saskatchewan Canada

**Keywords:** acid detergent fibre, *Brassica napus*, canola, EMS, haploid mutagenesis, oil, protein, structural variation

## Abstract

A key challenge for the genetic improvement of canola ( *Brassica napus* ), one of the world's most important oilseeds, is the limited natural variation for commercially important traits. The creation of new variation is hindered by the lack of functional knowledge about genes controlling these traits. Ploidy and genomic duplications in canola complicate the effective transfer of functional insights from Arabidopsis. Here, we report a novel functional genomics platform for rapid gene/trait discovery and optimisation. We established a double haploid population of 1240 lines from EMS‐mutagenised microspores of the spring‐type canola line, NRCDH4079. A platinum‐quality reference genome, gene annotations and a gene expression atlas from developing seeds were generated for NRCDH4079. Exome sequencing of the mutagenised population resulted in the development of a ‘TILLED’ database, revealing 1243 premature stop codons across 1222 genes, along with 140 522 moderate‐effect or modifier variants impacting 70 626 genes. Phenotypic analysis revealed significant variation in key seed traits, including oil, protein and acid detergent fibre (ADF). Notably, the mutant variant DP125410314 exhibited increased protein and reduced ADF, two important traits for improving the meal composition of canola. Genetic mapping of this variant identified a homoeologous non‐reciprocal translocation between A1 and C1 chromosomes associated with reduced ADF content, highlighting the role of structural variations in trait development. This work establishes haploid mutagenesis as a powerful tool for crop improvement, with broader implications for other *Brassica* species. By enhancing our understanding of seed protein traits, it lays the foundation for canola varieties that meet future nutritional and market demands.

## 1 Introduction

Canola ( *Brassica napus* L.) is one of the most important oilseed crops worldwide. Global production of oilseed Brassica crops was estimated at 88 million tons in 2024 (https://www.fao.org/faostat/en/#data/QCL). Canada is the world's largest producer (single country) of canola, accounting for approximately 21% of global canola production (USDA). First developed in the 1970s through the selective breeding of rapeseed, the name ‘canola’ is an acronym for ‘Canadian oil, low acid’. This name was chosen to distinguish canola from traditional rapeseed, which contained high levels of erucic acid and glucosinolates, compounds that make rapeseed oil and meal less desirable for human and animal consumption, respectively. Canola oil is characterised by having less than 2% erucic acid in its fatty acid profile, with the defatted solid component (meal) containing less than 30 μmol of glucosinolates per gram (Canadian Food Inspection Agency, 2017; http://inspection.canada.ca/en/plant‐varieties/plants‐novel‐traits/applicants/directive‐94‐08/biology‐documents/brassica‐napus).

Most of canola's economic value comes from its oil, which comprises about 45% of the seed (Daun 2007). Canola meal is a valuable source of protein for animal and poultry feeding and to produce protein concentrate and isolate for higher value feed and food uses (Wanasundara 2011). However, the nutritional value of canola meal is limited by lower levels of crude protein and higher levels of fibre than are present in soybean meal, and the presence of antinutritional compounds, such as glucosinolates, phytic acid and tannins, which can affect nutrient absorption and animal performance (Khajali and Slominski 2012; Mejicanos et al. 2016). Acid Detergent Fibre (ADF) is a major component of canola meal fibre and plays a critical role in determining its nutritional value. ADF primarily consists of cellulose and lignin, the most indigestible parts of the plant cell wall. The high fibre content of canola meal can limit its digestibility and reduce its energy concentration (Montoya and Leterme 2009). While ADF is not a primary target in most breeding programs, emerging genetic studies are beginning to explore its role in improving meal quality. Efforts to reduce ADF are gaining attention for their potential to enhance both feed quality and economic returns.

Availability of genetic variation for desirable seed components is the foundation for sustainable development of canola hybrids with high (quality and quantity) protein, and reduced fibre and antinutritionals, for new, higher‐value markets. Breeders take advantage of the natural genetic variation that is generated by spontaneous mutations, random meiotic assortment and recombination of linkage groups and alleles to form novel haplotypes. However, intensive breeding and continuous selection within elite, adapted germplasm for high yield and seed oil has led to narrowing of the genetic base of the crop (Fu and Gugel 2010; Rahman 2013), particularly with respect to meal‐related traits. Supplementing traditional prebreeding with approaches such as mutation breeding can rapidly broaden useful genetic variation available to breeders (Ahloowalia et al. 2004; Havlickova et al. 2024; Jung and Till 2021; Tang et al. 2020). To realise the compositional improvements needed to make canola a more valuable source of protein, it is critical to generate usable genetic diversity for protein‐related traits (quality and content) and advance our understanding of the genetic and functional components that regulate the biosynthesis of seed protein, fibre and antinutritional components.

Haploid mutagenesis is a powerful genetic tool that can be used to create novel genetic variations in plants (Ferrie and Möllers 2011). It involves the induction of mutations in haploid plant cells, such as microspores, which can then be regenerated into homozygous doubled haploid (DHs) plants in a single generation (Ferrie et al. 2008). Combining mutagenesis with the doubled haploid technique simplifies and accelerates the detection of recessive mutations and phenotypic evaluation. Canola microspores, being haploid and highly responsive to embryogenesis, are particularly well‐suited for mutagenesis, as any induced genetic changes are consistently expressed in the regenerated plants and their progeny, eliminating issues of heterozygosity and chimerism. Thus, haploid mutagenesis can accelerate the breeding process by reducing the time required to develop new trait sources, confirm their value and deploy them commercially. There are several strategies for inducing mutations in haploid plant cells, including chemical mutagenesis, irradiation and transposon insertion. Ethyl methane sulfonate (EMS), a chemical mutagen that induces high‐frequency point mutations and small INDELs (insertions and deletions), has become one of the most widely used mutagens in plants (Kim et al. 2006).

Mutagenesis of microspores has been used in canola in the past for oil content and quality, but selection was based only on phenotype (McClinchey and Kott 2008; Swanson et al. 1989). Here, we describe the establishment of a doubled haploid population of 1240 lines derived from EMS‐mutagenised microspores of the spring‐type canola line NRCDH4079. The entire population is genotyped using an exome capture array and phenotyped in the field to identify lines with increased protein content and reduced fibre and antinutritional compounds such as glucosinolates. Using a linkage mapping strategy, we identify a homoeologous non‐reciprocal translocation between chromosomes A1 and C1 associated with reduced ADF levels. This work establishes a foundational resource for future trait characterisation, genetic mapping and improvement of canola meal quality.

## 2 Results

### 2.1 Generating Mutagenised Doubled Haploid Populations Through Microspore Mutagenesis

The *Brassica napus* genotype NRCDH4079, a DH line derived from the Swedish spring‐type cultivar Topas, was selected for mutagenesis due to its high embryogenic potential (Ferrie and Möllers 2011). Following an established haploid mutagenesis protocol (Ferrie et al. 2008), microspores of NRCDH4079 were treated with EMS at concentrations ranging from 0.1% to 0.4% (Figure S1). This approach generated 2583 haploid and DH plants, and flow cytometry analysis identified 1240 diploid/DH lines that successfully produced seeds. Among these, 669 lines were obtained from the 0.1% EMS treatment, 231 from 0.2%, 324 from 0.3% and only 34 from the 0.4% EMS concentration. As the EMS concentration increased, plant survival rates declined, resulting in the recovery of only 34 seed‐producing DH lines at the highest concentration (0.4% EMS) (Figure S2 and Table S1).

A broad spectrum of phenotypic variation was observed among the mutant DH lines. Some lines exhibited delayed bolting yet remained fertile, while others flowered significantly earlier. Several variants displayed sterility or reduced fertility, along with morphological deviations such as extreme dwarfism or excessive height. Additionally, diverse pod phenotypes were identified, including twin pods, fused pods and pedicel fusion. Seed coat pigmentation varied, ranging from green to light and dark brown, in contrast to the black‐seeded parental line (Figure S3). With the lowest EMS treatment, overall flowering time decreased compared with the control samples (Figure S4 and Table S2). As EMS concentration increased, flowering time initially rose and then declined again. At the highest EMS concentration (0.4%), flowering time did not differ significantly from that observed at 0.2% EMS. This easily observable phenotypic variation suggests that EMS‐induced microspore mutagenesis effectively generates a wide array of trait variants. This mutant population provides a valuable genetic resource for functional genomics, trait discovery and breeding applications in *B. napus* .

### 2.2 Genome Assembly and Annotation of *B. napus* Genotype NRCDH4079, the Parental Line Used for Mutagenesis

**FIGURE 1:**
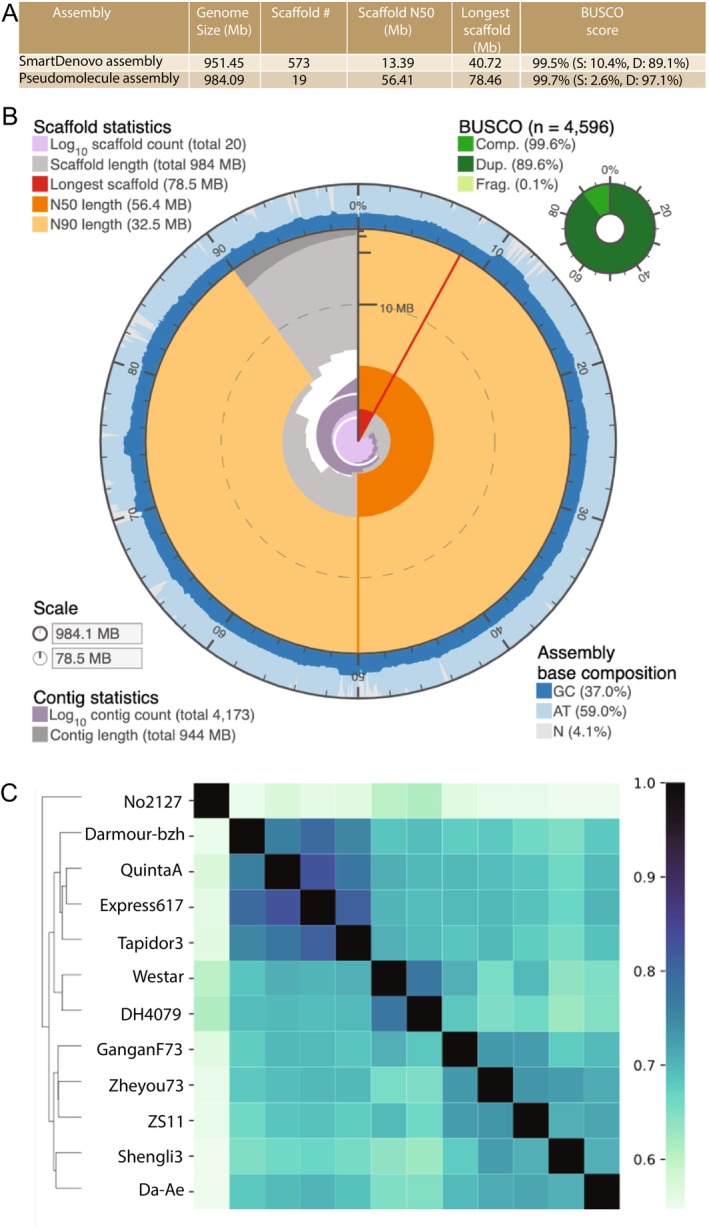
Genome assembly and comparative analysis of *B. napus* genotype NRCDH4079. (A) Summary statistics of genome assembly steps, including draft assembly using SmartDenovo followed by pseudomolecule construction through Hi‐C and BioNano‐based scaffolding. (B) Snail plot illustrating genomic features of the assembled genome. Scaffold count is represented in light grey (log_10_ scale) at the center, while scaffold lengths are shown in dark grey. The longest scaffold is highlighted in red along with its assembly coverage percentage. N50 and N90 values are marked with dark and light orange blocks, respectively. GC and AT contents are displayed as dark and light blocks on the outer ring, and total assembly length is indicated on the outermost scale. BUSCO completeness is presented in the top‐right corner. (C) Heatmap showing shared gene families (PanKmer analysis) between NRCDH4079 and other *B. napus* genotypes. Darker colours indicate a higher proportion of shared genes, reflecting greater genetic similarity.

A robust mutation discovery process requires a high‐quality reference genome of the mutagenised line NRCDH4079. Using existing *B. napus* reference genomes would lead to extensive false mutation calls due to genetic differences between the reference and the donor line. Therefore, we generated a chromosome‐scale genome assembly for NRCDH4079. Using Oxford Nanopore Technology (ONT), we obtained 91 gigabases (Gb) of long‐read sequencing data (Table S3). After stringent quality filtering, 79 Gb (~78× coverage) were assembled using SmartDenovo (Liu et al. 2021), producing 573 contigs spanning 951.45 megabases (Mb) with an N50 of 13.39 Mb (Figure 1A and Table S4). The final assembly had a GC content of 37% and was composed of 4.1% gaps (Figure 1B). Three rounds of polishing with Medaka and Racon (see Methods), incorporating both ONT and Illumina short‐read data (2 × 250 bp paired end reads; Table S4), refined the assembly to 571 contigs (N50 = 11.99 Mb). Scaffolding with BioNano genome maps further improved the assembly, yielding 54 super‐scaffolds that is, 2–3 per chromosome, enabling their ordering and orientation into 19 pseudomolecules and expanding the genome to 991.60 Mb (Tables S4 and S5). The final genome assembly spans 984.10 Mb, with 96.4% of the sequence assigned to chromosomes (Figure 1B). The integrity of the final genome assembly was verified with Hi‐C data. The assembly demonstrates high contiguity and completeness, capturing 99.70% of genes from the Benchmarking Universal Single‐Copy Orthologs (BUSCO) Brassicales dataset (Figure 1B), and the genome statistics are comparable to or are better than publicly available *B. napus* genome assemblies (Table S4). Mapping rates for Illumina and ONT reads (Table S3) exceeded 98.38% and 91.34%, respectively, while 91.7% of RNA‐seq reads aligned to the genome, confirming assembly accuracy.

To enhance gene annotation, Illumina RNA‐Seq data (2 × 125 bp paired end) were generated from 18 tissues spanning key developmental stages (Cotyledon, Flower, Inflorescence, Second Leaf, Fourth Leaf, Mature Leaf, Senescing Leaf, Root, Seed Development Day 4, Seed Development Day 8, Seed Development Day 12, Seed Development Day 16, Seed Development Day 20, Seed Development Day 24, Seed Development Day 30, Seed Development Day 36, Mature Seed and Sprout; Table S6). Ab initio gene prediction identified 149 246 genes, categorised into 115 296 high‐confidence and 33 950 low‐confidence genes based on proteomic datasets, pFam domains and assembled transcriptome data. Gene Ontology (GO) annotation assigned 1.49 million GO terms, with 99.9% of the genes annotated. Among 8098 unique GO terms, classifications included Biological Process (54.1%), Molecular Function (33.8%) and Cellular Component (12.0%). KEGG Orthology (KO) annotation via GhostKOALA (Kanehisa et al. 2016) identified 38 355 genes (25.7%) linked to 4334 unique KO IDs, spanning 426 KEGG pathways—surpassing the 4026 KO IDs mapped in the Darmor‐bzh genome. Additionally, 6791 transcription factors (TFs) were identified, with bHLH as the most abundant family, followed by AP2/ERF, C2C2, NAC, Homeobox, MYB and B3 (Figure S5). TF family representation was consistent with the Darmor‐bzh genome (Ghorbani et al. 2020; Ke et al. 2020; Wang et al. 2018).

Repetitive sequences, including both transposable elements (TEs) and tandem repeats (TRs), constituted approximately 58% of the NRCDH4079 genome (Table S7), a proportion consistent with repeat fractions reported for the recent gap‐free *B. napus* cv. Xiang5A assembly (Li et al. 2023). This close agreement underscores the high contiguity, completeness and repeat‐resolution quality of our assembly and annotation. The repeat landscape of NRCDH4079 was dominated by transposable elements, which together accounted for roughly 50% of the genome. Among retrotransposons, long terminal repeat (LTR) elements formed the largest group, with Gypsy elements (13.24%) representing the most abundant superfamily, followed by Copia (9.74%), TRIMs (1.80%) and unclassified LTRs (4.54%). Non‐LTR elements contributed an additional 3.54% from LINEs and 0.32% from SINEs (Table S7). DNA transposons were also prevalent, with terminal inverted repeat (TIR) elements comprising a major portion of this category. CACTA elements accounted for 5.81%, followed by MuDR (4.01%), PIF (1.56%), hAT (1.95%) and Tc1 (0.48%). MITEs occupied 0.84% of the genome. Additionally, non‐TIR Helitrons contributed 5.27% of the assembly (Table S7). Furthermore, full‐length LTR analysis identified 4777 Copia LTRs—predominantly Ale (2250), Bianca (1070) and 3852 Gypsy LTRs, mainly from the Athila (1147) and CRM (1164) subfamilies (Figure S6). Approximately 75% of LTR families proliferated within the last 1 million years (MY), with Copia Ale LTRs undergoing the fastest expansion, forming 98% of LTRs within the past 2 MY (Figure S7). Genome‐wide analysis identified 4293 Miniature Inverted‐repeat Transposable Elements (MITEs) across five superfamilies, with Tourist (2310), hAT (955) and CACTA (923) as the most abundant (Figure S8). Although MITEs lack autonomous transposition ability, they significantly influence gene regulation, genetic diversity and genome evolution (Chen et al. 2012). Notably, 38% of MITEs were located within < 1 kb of genes, suggesting a potential regulatory role. Centromeric regions were also well‐assembled, with 3% of sequences corresponding to centromeric repeats, including abundant Ale LTRs (Table S8), consistent with previous findings (Perumal et al. 2020). The genome assembly and annotation are available at https://bioinfo.nrc.ca/tilling/downloads.html.

A comparative analysis using PanKmer (Aylward et al. 2023) evaluated NRCDH4079's genome relative to other *B. napus* genomes. Results showed, not surprisingly, the highest similarity to Westar, another spring canola variety (Figure 1C). Other genomes clustered into two groups: semi‐winter cultivars (e.g., GanganF73, Zheyou73, ZS11, Shnegi3, Da‐Ae) and winter cultivars (e.g., Darmor‐bzh, QuintaA, Express 617, Tapidor3) (Figure 1C).

### 2.3 *B. napus* Exome Capture Array

To identify and characterise EMS‐induced mutations in the DH population, we employed an exome capture array developed by Dr. Isobel Parkin for the DH12075 genome, a Canadian spring canola cultivar (https://cruciferseq.ca). Designed in collaboration with Roche NimbleGen, this array targeted all 105 791 protein‐coding genes in DH12075, encompassing 1 785 711 capture targets that densely tiled gene exons, covering 149 702 345 base pairs (bp). The array achieved 96.1% coverage of the DH12075 exome, with an estimated total capture size of 198 262 530 bp (Table S10). Similarly, the array captured 95.52% of the NRCDH4079 exome, with only 6674 genes not represented, most of which were low‐confidence genes (Table S10).

### 2.4 Re‐Sequencing the Mutant Population via Exome Sequencing

**FIGURE 2:**
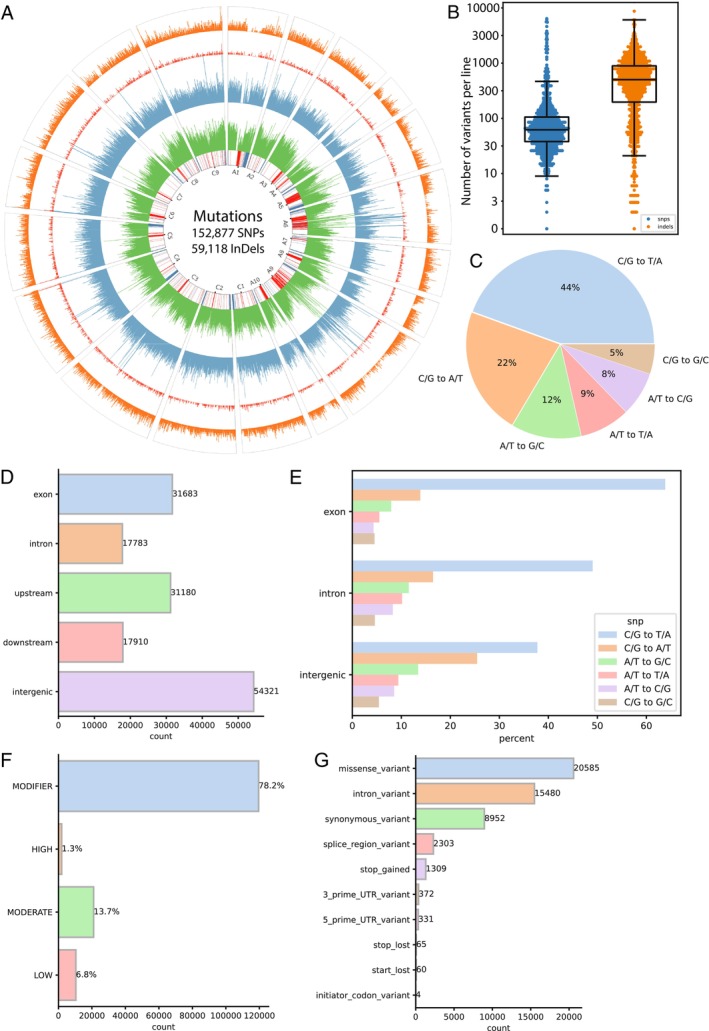
Genome‐wide distribution and functional annotation of EMS‐induced variants in 1240 mutant DH lines. (A) Circos plot displaying the genome‐wide density of 152 877 SNPs and 59 118 insertions and deletions across chromosomes, binned at 100 kb intervals. The innermost track represents chromosomes with the distribution of tandem repeats. Outward tracks illustrate gene density, single nucleotide polymorphism (SNP) coverage, insertion coverage, and deletion coverage, respectively. (B) Distribution of the total number of variants (SNPs and InDels) per DH line. Y‐axis is shown on a logarithmic scale. (C) Pie chart depicting the types and relative frequencies of EMS‐induced SNP mutations. (D) Genomic distribution of EMS‐induced SNPs across exon, intron, upstream (2 kb), downstream (2 kb), and intergenic regions. (E) Proportional frequency of different mutation types observed within exon, intron, and intergenic regions. (F) Classification of SNPs based on predicted functional impact: high, moderate, low and modifier. (G) Functional annotation of mutation effects on genes, including the density of affected gene functions.

Exome captures from 1240 individually indexed EMS‐mutagenised NRCDH4079 DH lines were multiplexed and sequenced on the Illumina platform, generating 13.62 Tb of data (Table S11) and enabling accurate recovery of mutation profiles for each line. Each mutant sample yielded an average of 11.83 Gb, achieving 30X exome coverage. After alignment of this sequencing data to the NRCDH4079 reference genome, variant calling and filtering, 211 995 variants were identified, including 152 877 SNPs and 59 118 insertions and deletions (InDels; Figure 2A and Table S12). The average mutation density was 155 SNPs/Mb and 60 InDels/Mb. The number of mutations per mutant ranged from 2 to 6414 SNPs and 1 to 12 988 InDels per mutant line (Figure 2B). In contrast, non‐mutagenised controls had only 49 SNPs per plant, confirming a low error rate. Given the smaller number of genes in DH12075, on which the exome array was based, the number and density of detected variants in each line may be slightly underestimated.

EMS‐induced mutations were predominantly C/G to T/A transitions (44.48%), followed by C/G to A/T (22.02%), A/T to G/C (12.07%), A/T to T/A (8.67%), A/T to C/G (7.63%) and C/G to G/C (5.14%) (Figure 2C and Table S12). Based on genome annotations, 31 683 SNPs were in exons, 17 783 in introns, 31 180 in upstream (2Kb), 17 910 in downstream (2Kb) and 54 321 in intergenic regions (Figure 2D). C/G to T/A transitions were most frequent in exons (64%), followed by introns (49%) and intergenic regions (38%) (Figure 2E).

When examining the average number of SNPs per sample across EMS treatments, no consistent trend was observed. Although there was a statistically significant difference between the 0.1% and 0.3% EMS groups, this pattern did not extend across increasing EMS concentrations (Figure S9). In contrast, the average number of indels showed a clear and statistically significant increase with each stepwise increase in EMS concentration (Figure S10). These findings indicate that, while SNP frequency does not correlate strongly with EMS dosage, indel frequency displays a robust positive correlation with increasing EMS concentrations.

Functional effect prediction using SnpEff (Cingolani et al. 2012) classified mutations into 1.3% high‐impact (e.g., nonsense, frameshift mutations), 13.7% moderate‐impact (missense mutations), 78.2% modifier‐impact (intronic, intergenic mutations) and 6.8% low‐impact (synonymous mutations) (Figure 2F). A total of 1243 premature stop‐gain events were identified, affecting 1222 non‐redundant genes. Further analysis identified 20 585 missense variants, 8952 synonymous variants, 17 783 intron variants, 2303 splice‐region variants, 65 stop‐lost mutations, 60 start‐lost mutations and 4 initiator codon variants (Figure 2G).

To ensure that there were no SNP biases towards the sub‐genomes, we analysed the types of SNPs, their locations and their impacts across the CC and AA sub‐genomes. The size difference between the two sub‐genomes was taken into account, and there was no discernible bias or anomalies in the distribution of SNP type, impact or location (Table S13).

### 2.5 Reverse Genetics: Identification of EMS‐Derived Mutations in Acyl‐Lipid Metabolism Genes

**FIGURE 3:**
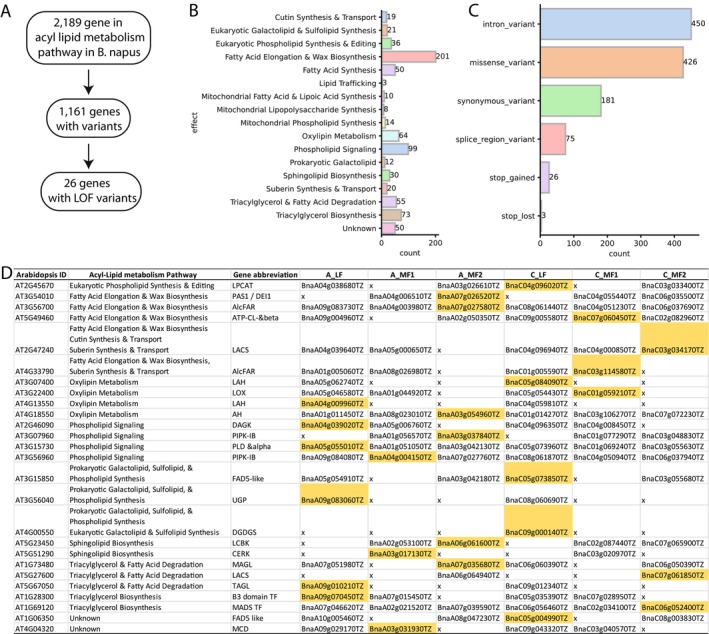
Reverse genetic analysis of acyl‐lipid metabolism‐related genes in 1240 mutant DH lines. (A) Overview of acyl‐lipid metabolism‐related genes in the NRCDH4079 genome, comprising 2189 genes. Among these, 1161 genes contained SNP variants in the mutant DH lines, with 26 genes identified as carrying premature stop‐gain mutations. (B) Pathway annotation of genes with variants, highlighting the density and distribution of affected pathways involved in acyl‐lipid metabolism. (C) Functional annotation of the predicted effects of mutations on acyl‐lipid metabolism pathway genes, including the density of affected gene functions. (D) Detailed representation of acyl‐lipid metabolism pathway genes carrying premature stop‐gain mutations. Affected pathways are shown along with their corresponding Arabidopsis orthologs and NRCDH4079 homoeologs in the *B. rapa* and *B. oleracea* sub‐genomes, classified into least fractionated (LF) and most fractionated (MF1 and MF2) categories. Genes with premature stop‐gain mutations are highlighted in yellow.

Reverse genetics enables the functional characterisation of genes through targeted approaches, with EMS‐induced mutagenesis being a robust method for generating allelic variants linked to specific genomic loci. To demonstrate the utility of our TILLING platform in reverse genetics, we analysed EMS‐induced mutations in genes involved in acyl‐lipid metabolism pathways (Li‐Beisson et al. 2013). For this purpose, we constructed a syntelog matrix, which pairs *A. thaliana* genes with their homoeologs in the NRCDH4079 genome (Supporting Information 1). The enumeration of genes implicated in acyl‐lipid metabolism (Li‐Beisson et al. 2013) revealed 2189 non‐redundant NRCDH4079 genes associated with various stages of lipid biosynthesis, accumulation and degradation (Figure 3A). Of these, 1161 genes, which play roles in different stages of acyl‐lipid metabolism (Figure 3B), contained high‐ and moderate‐impact variants (Figure 3C). Notably, our analysis uncovered 26 stop‐gained mutations across 23 distinct genes related to acyl‐lipid metabolism (Figure 3D). Assessment of the seed oil, seed protein, meal protein and meal fibre content (BLUEs) in lines carrying premature stop codons in acyl‐lipid pathway genes showed no significant differences from the parent NRCDH4079 or the non‐mutagenised control lines for any of the four traits (Table S14). As most oil traits are likely controlled by multiple loci, it will be necessary to combine mutations to observe significant effects.

### 2.6 Forward Genetics: Genetic and Phenotypic Characterisation of Mutants for Seed Compositional Traits in Canola

Forward genetics remains a powerful approach for functional genomics in plants, complementing reverse genetics. To demonstrate the utility of the mutant collection for gene isolation in ‘canola’, we developed an efficient, cost‐effective pipeline that integrates both approaches. This strategy was applied to identify genetic determinants of low ADF in seed composition mutants.

#### 2.6.1 Field Phenotyping of the Mutant and Mapping Populations

##### 2.6.1.1 Mutant Population

A total of 1200 double haploid mutant lines out of 1240 that produced sufficient seeds were field‐grown at two locations, and seed compositional traits were assessed using near‐infrared reflectance spectroscopy (NIRS; Table S15). The NIRS predictive equations used in this study were developed by the Corteva Agriscience PCA lab in Eschbach. These models are built using internal samples collected from diverse research field locations and measured on a FOSS XDS instrument across a spectral range of 400–2500 nm. Reference values for all parameters were obtained via wet chemistry analyses. The models are updated annually to maintain accuracy and robustness. The coefficients of determination (*R* ^2^) for the calibration models were as follows: seed oil (0.97), seed protein (0.98), meal protein (0.89) and ADF (0.91). Best Linear Unbiased Estimates (BLUEs) for seed oil, seed protein, meal protein and meal acid detergent fibre (ADF) were derived from the NIRS data (Table 1 and Table S16). The elite inbred line (CHECK) was included as a comparator, given that the NRCDH4079 donor genotype, which was mutagenised to generate the mutant population, represents older genetics. Mean trait values, population ranges and standard errors of differences for all four traits are summarised in Table 1.

**TABLE 1:** BLUE means from analysis for donor (NRCDH4079), mean of mutant population (Pop Avg), and elite comparator (CHECK) for four compositional traits.

Name	Seed oil	Seed protein	Meal protein	Meal ADF
CHK	47.6	24.6	46.8	16.2
NRCDH4079	45.9	26.2	48.5	18.6
Pop Avg	45.8	26.9	49.5	18.3
SED	1.032	1.016	1.136	0.584
Range	41.5–48.7	24.0–30.9	46.3–54.3	14.2–20.0

*Note:* The range for each trait and standard error of difference (SED) are also shown.

**FIGURE 4:**
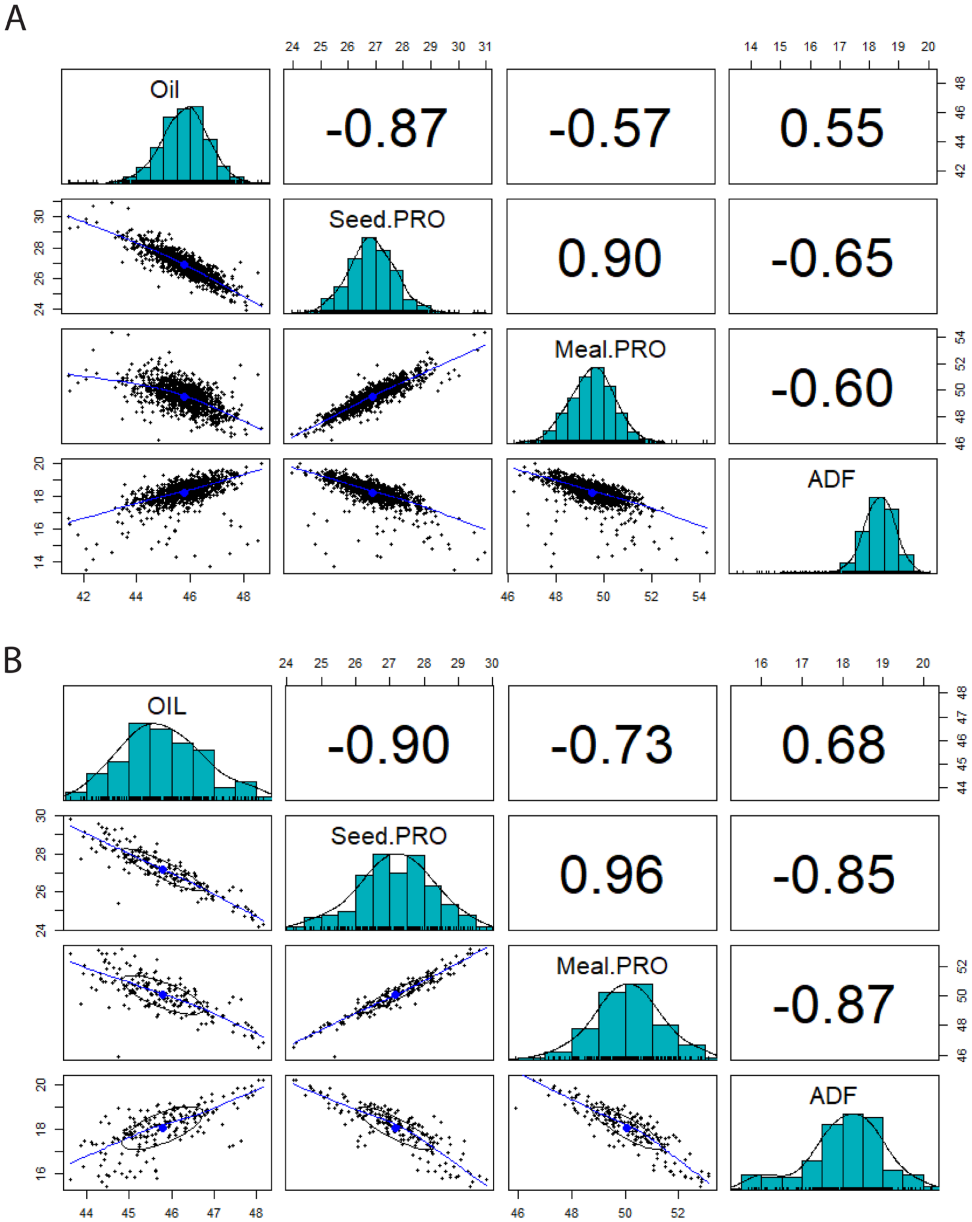
Correlation and distribution of four compositional traits (Oil, seed protein, meal protein and meal ADF) in mutant and mapping populations. (A) Histograms, scatter plots and correlation coefficients for four compositional traits in the 1240 mutant DH lines. (B) Histograms, scatter plots and correlation coefficients for four compositional traits in the mapping population derived from the cross between NRCDH4079 and DP125410314.

While population means did not significantly differ (*p* < 0.05) from the NRCDH4079 donor for any trait, the mutant population exhibited extensive variation, with ranges of 7.2 percentage points (ppt) for seed oil, 6.9 ppt for seed protein, 8.0 ppt for meal protein and 5.8 ppt for meal ADF. Individual lines with extreme values (both highest and lowest) and many lines close to these individuals differed significantly from the population mean (*p* < 0.05) and the NRCDH4079 donor, suggesting that mutagenesis successfully introduced novel genetic variation. Histograms, scatter plots and correlation coefficients for the four traits are presented in Figure 4A. Seed protein and meal protein were positively correlated, while both were negatively correlated with seed oil and meal ADF. Seed oil and meal ADF exhibited a positive correlation.

Based on these findings, mutant line DP125410314, which displayed the lowest meal ADF, was selected as a parent for developing the mapping population.

##### 2.6.1.2 Mapping Population

To investigate the genetic basis of seed compositional traits, with specific focus on meal ADF, a mapping population was created by crossing DP125410314 with the NRCDH4079 donor. *F*_1_ plants were self‐pollinated to generate *F*_2_ seeds, and 200 *F*_2_ plants were grown in a greenhouse and self‐pollinated to produce a mapping population of 184 *F*_3_ lines. DNA samples were collected from *F*_2_ plants for genotyping, and F_3_ lines representing each family (see Methods) were field‐grown at two locations in 2022 to generate seed for NIRS‐based trait data collection (Table S17).

The parents of the mapping population only differed significantly for meal ADF (Table 2). BLUE values for seed oil, seed protein, meal protein and meal ADF in the mapping population (Table 2 and Table S18) revealed that population means for seed oil and seed protein did not differ significantly (*p* < 0.05) from either parent. However, population means for meal protein levels were significantly lower (*p* < 0.05) than both parents, while meal ADF values were higher than those of DP125410314 but not significantly different from NRCDH4079.

**TABLE 2:** BLUE means from analysis for donor (NRCDH4079) and mutant (DP125410314) parents, the mid‐parent value, and the mean of the mutant population (Pop Avg) for four compositional traits.

Name	Seed oil	Seed protein	Meal protein	Meal ADF
NRCDH4079	44.0	29.2	52.0	17.5
Mutant Parent	44.4	29.4	52.9	15.7
Mid‐parent	44.2	29.3	52.5	16.6
Pop Avg	45.8	27.1	50.0	18.1
SED	1.908	1.234	0.905	0.882
Range	43.6–48.2	24.2–29.8	45.9–53.1	15.4–20.2

*Note:* The range for each trait and standard error of difference (SED) are also shown.

The mapping population displayed substantial variation for all four traits, with observed ranges of 4.6 ppt for seed oil, 5.6 ppt for seed protein, 7.2 ppt for meal protein and 4.8 ppt for meal ADF. Histograms, scatter plots and correlation coefficients (Figure 4B) demonstrated patterns similar to the mutant population, with strong negative correlations between seed oil and seed protein, as well as meal ADF. Seed protein and meal protein were positively correlated, while meal protein and meal ADF exhibited a strong negative correlation.

Table 3 summarises the coefficients of variation (CV) and broad‐sense heritability estimates (*H* ^2^) for the four traits in both populations. CV values were comparable between the mutant and mapping populations and heritability estimates for seed oil and meal ADF were consistent across populations. However, *H* ^2^ values for seed protein and meal protein were significantly higher in the mapping population compared to the mutant population. For meal ADF, the *H* ^2^ estimate obtained here is in the range reported previously (Gacek et al. 2021; Miao et al. 2019; Yusuf and Möllers 2024). The F_3_ lines in the mapping population with the lowest meal ADF had ADF levels similar to the mutant parent and significantly lower than both the NRCDH4079 parent and the mutant population mean, confirming that a heritable variant for reduced meal ADF was created through mutagenesis.

**TABLE 3:** Coefficient of variation (CV) and broad‐sense heritability estimates (*H* ^2^) for four compositional traits measured in the mutant population and the mapping population.

Trait	Mutant population		Mapping population	
	*H* ^2^	CV	*H* ^2^	CV
Seed oil	0.18	3.5	0.16	3.2
Seed protein	0.10	6.3	0.31	6.0
Meal protein	0.11	3.9	0.35	3.7
Meal ADF	0.44	7.7	0.55	6.1

### 2.7 A Homoeologous Non‐Reciprocal Translocation Reduces Meal Fibre and Enhances Protein Content in DP125410314

**FIGURE 5:**
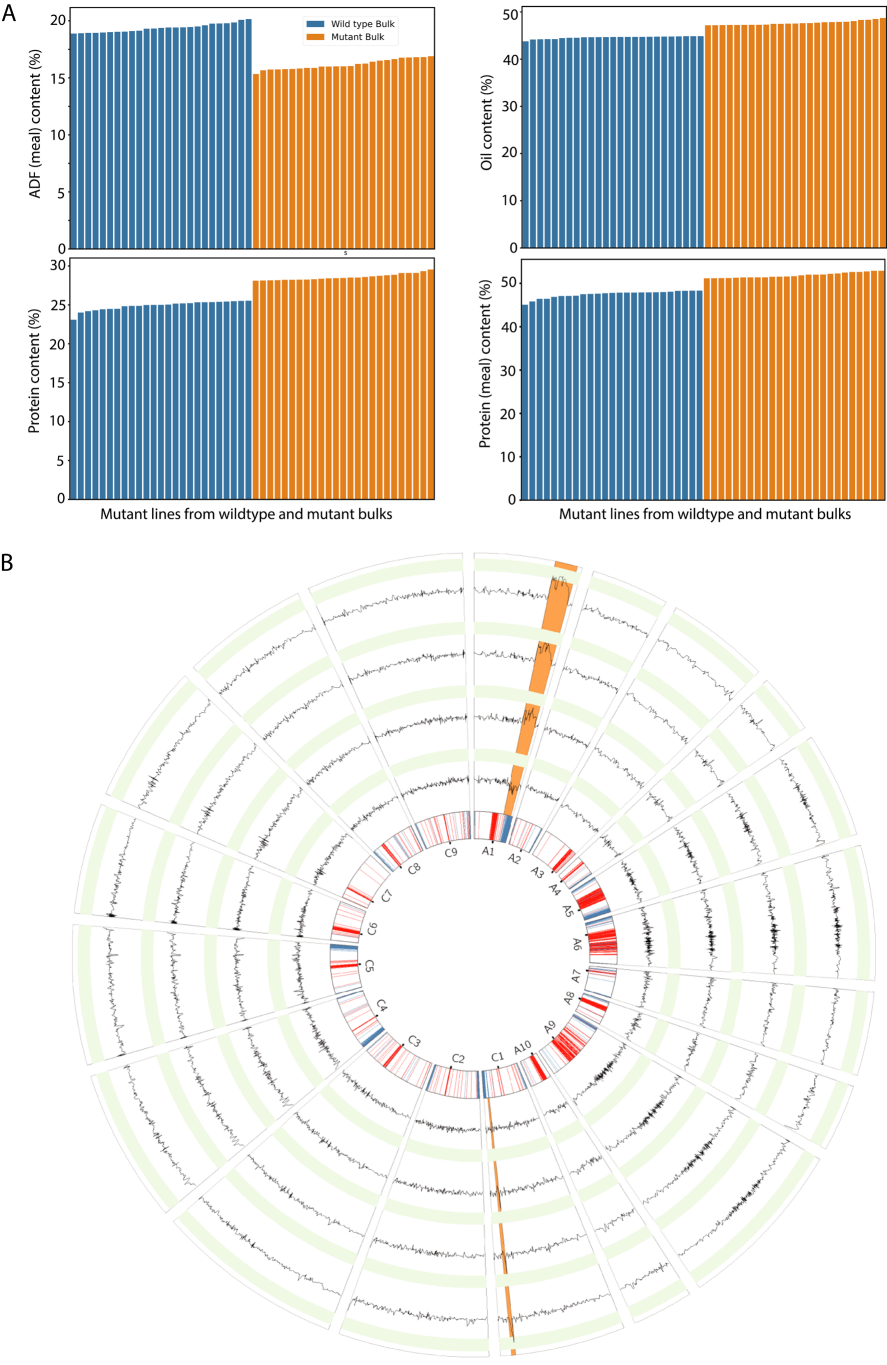
Identification of causal variants for compositional traits using the MutMap strategy in mutant line DP125410314. (A) Distribution of four compositional traits, including meal ADF content, oil, seed protein and meal protein in 25 selected lines forming wild‐type and mutant bulks for each trait. The bulks were derived from the mapping population obtained from a cross between NRCDH4079 and DP125410314. (B) ΔSNP‐index plots generated by subtracting SNP indices of the wild‐type bulk from the mutant bulk. Dark lines represent sliding averages calculated over 5 SNPs. Shaded regions on chromosome A1 and C1 indicate genomic intervals where the ΔSNP‐index exceeds 0.9, representing statistically significant differences between bulks. The innermost track displays chromosomes with tandem repeat distributions, while the outer tracks (from inner to outer) correspond to ΔSNP‐index plots for oil, seed protein, meal protein and meal ADF, respectively.

To identify genomic regions associated with the low meal ADF and high meal protein phenotypes, shotgun sequencing (~8× coverage per sample) was performed on DNA from F_2_ individuals. NIRS‐based trait data were used to select 25 *F*_2_ progenies with the lowest meal ADF and highest meal protein (Fmut, Pmut) and 25 progenies with the highest meal ADF and lowest meal protein (Fref, Pref) (Figure 5A). Sequence reads from these groups were combined into pseudo‐bulks, aligned to the NRCDH4079 reference genome and analysed for single‐nucleotide polymorphisms (SNPs). SNP indices for each variant were calculated and plotted across all 19 chromosomes (Figure 5B). The use of extreme‐phenotype bulks for whole‐genome sequencing follows the well‐established MutMap/BSA‐seq strategy (Abe et al. 2012) and provides a rapid and effective means of identifying genomic regions associated with complex traits.

As expected, SNP indices were randomly distributed around 0.5 across most of the genome. However, specific genomic regions on chromosomes A1 and C1 displayed clusters of high‐SNP‐index variants (> 0.9), spanning 11.16 Mbp and 3.95 Mbp, respectively (Figure 5B). These regions contained an average of 0.97 SNPs per gene, totalling 119 high‐confidence SNPs likely associated with frameshift mutations or deletions. Notably, the same regions harboured SNP clusters associated with high meal protein content, suggesting that these loci contribute to the observed phenotypic variation. To identify the causal SNPs for the low meal ADF and high meal protein traits, we focused on variants with a SNP index > 0.9 within the A1 and C1 regions. Notably, most A1‐region SNPs were concentrated within a narrow segment, and none were classified as high‐impact or modifier variants.

**FIGURE 6:**
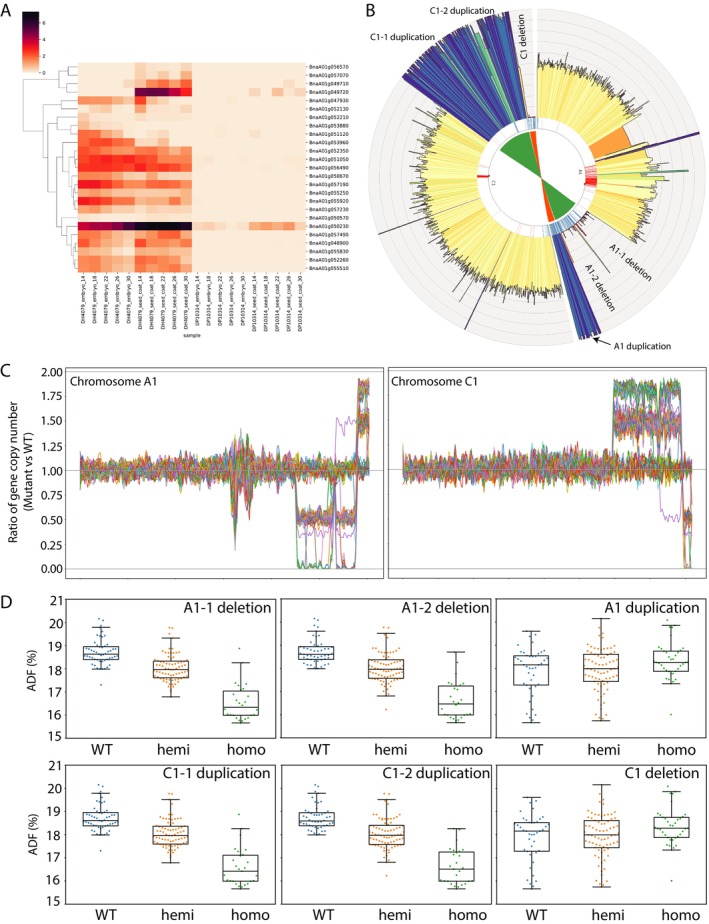
A homoeologous non‐reciprocal translocation between chromosomes A1 and C1 reduces meal fibre and enhances protein content in *Brassica napus* line DP125410314. (A) Expression profiles of differentially expressed genes located in the A1 genomic region associated with causal variants for meal ADF and meal protein content. RNA‐seq analysis was performed on developing seed samples from multiple embryo stages (E4–E7, corresponding to 14, 18, 22, and 26 days after anthesis, respectively) and their sub‐compartments (embryo and seed coat) in both DP125410314 and wild‐type plants. (B) Circos plot illustrating homoeologous non‐reciprocal translocations between chromosomes A1 and C1 within the region linked to meal ADF and meal protein traits. The innermost track displays chromosomes A1 and C1. The outer track represents sequencing read coverage from DP125410314, with blue peaks indicating duplicated regions and gaps denoting deletions. (C) Genome‐wide distribution of homoeologous non‐reciprocal translocations identified using LSV‐viz across 184 *F*_2_ progenies from the mapping population derived from the NRCDH4079 × DP125410314 cross. Duplication and deletion events were classified as hemizygous or homozygous based on the ratio of gene copy numbers in the mutant relative to the wild type. (D) Phenotypic analysis of 184 *F*_2_ progenies, grouped based on genotype as homozygous, hemizygous (deletion or duplication) or wild type, revealed a strong association between deletions on chromosome A1 and corresponding duplications on chromosome C1 with reduced meal ADF content.

To further investigate these genomic changes, transcriptome analysis was conducted using RNA sequencing of developing seed samples from multiple embryo stages (E4–E7) and their sub‐compartments (embryo and seed coat) in both DP125410314 and wild‐type plants. None of the differentially expressed genes within the A1 region were expressed in the mutant, suggesting a possible deletion (Figure 6A). Structural variant analysis using the LSV‐viz method (*see* Methods) revealed two large deletions within A1, interrupted by a small, retained segment, followed by a duplication (Figure 6B). Similar deletions and duplications were identified on C1, mirroring the structural changes in A1. The identification of SVs through the alignment of reads from DP125410314 to the parent genome sequence of NRCDH4079 confirmed their absence in the parental line. The NRCDH4079 reference genome is supported by a BioNano optical map and robust scaffolding, ensuring high structural accuracy. Given this strong genomic framework, the presence of the SVs in DP125410314 is unlikely to result from assembly errors. Furthermore, none of these structural variations were found in other *B. napus* lines with publicly available genome sequences, which further supports the hypothesis that the observed variations were induced by the EMS treatment or the DH protocol, rather than resulting from limitations in genome assembly.

Extending the LSV‐viz analysis to all 184 F_2_ progenies confirmed that the first two deletions in A1 co‐segregated, while the duplication segregated independently (Figure 6C). Detailed phenotypic comparison revealed that individuals carrying the A1 deletions consistently exhibited reduced meal ADF content, and this effect was further influenced in progenies also carrying the C1 duplications (Figure 6D). This strong genotype–phenotype correlation indicates that the initial two homoeologous non‐reciprocal translocations between A1 and C1 are the primary causal variants responsible for the reduced meal fibre phenotype in DP125410314.

Importantly, the LSV‐viz approach provides an advantage over bulked‐segregant (Mutmap) mapping by enabling single‐individual resolution. While contrasting bulks identify regions enriched for trait‐associated alleles, LSV‐viz captures the independent segregation and co‐occurrence of deletions and duplications, directly linking structural variants to phenotypic variation. The co‐occurrence of deletions and duplications may influence gene dosage or disrupt gene function, providing a likely mechanistic basis for the observed reduction in meal ADF content.

### 2.8 Candidate Genes Associated With Reduced ADF and Elevated Seed Protein Content in DP125410314

**FIGURE 7:**
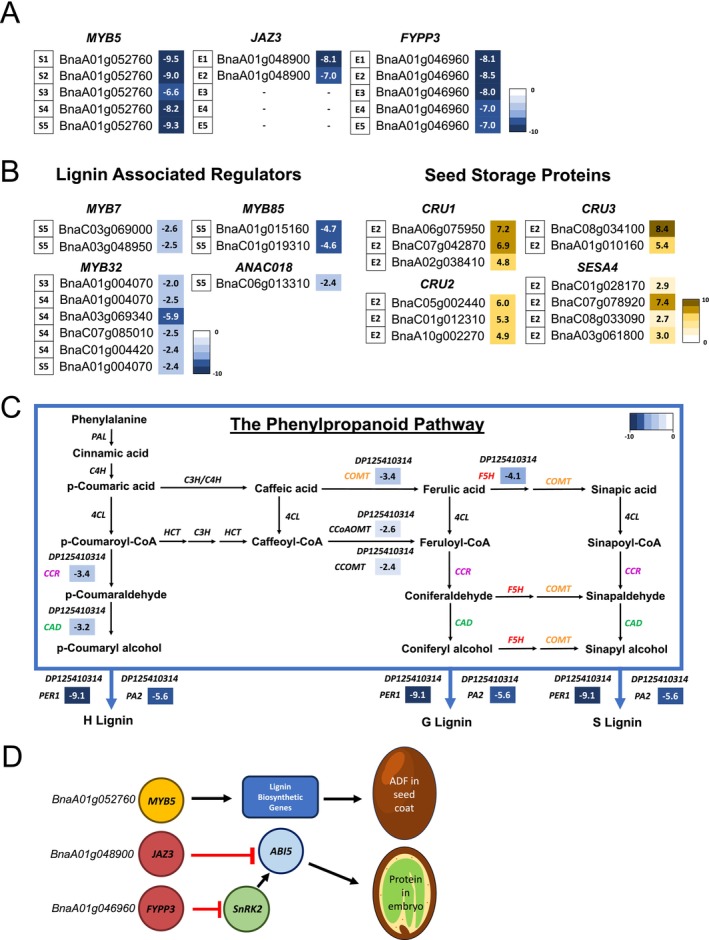
Deletion of *MYB5*, *JAZ3* and *FYPP3* on chromosome A1 is likely associated with high protein and low ADF in DP125410314. (A) Differential expression analysis of *MYB5*, *JAZ3* and *FYPP3*, located within the deleted A1 chromosomal region, identified these genes as potential causal regulators in the DP125410314 mutant. *MYB5* is associated with lignification, while *JAZ3* and *FYPP3* are implicated in seed storage protein accumulation. Expression profiles are shown for seed coat (S1–S5) and embryo (E1–E5) stages from DP125410314 compared to the NRCDH4079 control. (B) Expression patterns of lignin biosynthesis‐related transcription factors (*MYB7*, *MYB32*, *MYB85*, *ANAC018*) and seed storage protein genes (*CRU1*, *CRU2*, *CRU3*, *SESA4*) highlight altered regulatory networks in DP125410314. (C) Overview of the phenylpropanoid pathway showing genes that are significantly upregulated or downregulated in the DP125410314 seed coat relative to the NRCDH4079 control. Fold change values relative to wild type are shown only for differentially expressed genes. (D) Schematic pathway depicting normal seed development with proper *MYB5*, *JAZ3* and *FYPP3* function, and the resulting phenotypic changes in DP125410314 due to their downregulation or deletion, specifically reduced ADF and enhanced seed storage protein content.

To identify causal genes responsible for reduced ADF and enhanced seed protein content in DP125410314, we investigated genes located within the deleted (A1‐1/A1‐2)/duplicated (C1‐1/C1‐2) regions. Among the genes in the deleted region on chromosome A1, we identified *MYB DOMAIN PROTEIN 5* (*MYB5*) as a potential candidate associated with the reduced ADF phenotype. The gene copy of *MYB5* (BnaA01g052760) was severely downregulated or absent during seed coat development S1‐S5 in DP125410314 compared to the NRCDH4079 control (Figure 7A), suggesting that transcriptional regulation of target genes downstream of MYB5 may be altered in the mutant. Given that MYB5 is known to regulate lignin biosynthesis, we further examined changes in key regulators of the lignin biosynthetic pathway using RNA‐seq data. This analysis revealed downregulation of *MYB DOMAIN PROTEIN 7/32/85 (MYB7/32/85)* and *ARABIDOPSIS NAC DOMAIN CONTAINING PROTEIN 18 (ANAC018)*, particularly at seed coat developmental stages S3–S5 for MYB32 and S5 for *MYB7/85* and *ANAC018* (Figure 7B). This was accompanied by reduced expression of multiple phenylpropanoid pathway genes across seed coat developmental stages in DP125410314 (Figure 7C). These patterns are consistent with a potential reduction in lignin biosynthesis, which may contribute to the lower ADF levels observed in this line. However, further functional validation is required to confirm the roles of MYB5 and associated transcription factors in this phenotype.

We then assessed whether loss or duplication of genes could explain the enhanced protein content in DP125410314. Two genes, *JASMONATE‐INSENSITIVE 3 (JAZ3)* (BnaA01g048900) and *FLOWER‐SPECIFIC PHYTOCHROME‐ASSOCIATED PROTEIN PHOSPHATASE 3 (FYPP3)* (BnaA01g046960), located in the deleted A1 region, emerged as potential candidates. Both genes were strongly downregulated during embryo development stages E1–2 and E1–5, respectively (Figure 7A). Notably, both these genes have been implicated in repressing ABI5‐dependent pathways, which are involved in seed maturation and accumulation of seed storage proteins. Consistent with this possibility, we observed increased expression of several seed storage protein genes, including *CRUCIFERIN 1/2/3 (CRU1/2/3)* and *SEED STORAGE ALBUMIN 4 (SESA4)*, at embryo developmental stage E2 in DP125410314 (Figure 7B), coinciding with the downregulation or absence of *JAZ3*. These findings could suggest a potential link between loss of *JAZ3* and *FYPP3* expression and elevated seed protein content, though additional studies are needed to establish causality. Further research, including in‐depth genetic and functional characterisation of these candidate genes, is required to confirm their roles in controlling these traits.

### 2.9 *B. napus* TILLING Database

To enable broad access to identified variants, we developed the TILLED variant web database (https://bioinfo.nrc.ca/tilling/) using exome sequencing data from 1240 mutagenised lines. This resource provides a comprehensive genomic profile and offers true‐breeding lines for foundational and applied research.

The database catalogues 152 877 SNPs across the mutant population, allowing users to search variants by gene name or mutant line. Results are presented in a tabular format, with each SNP detailed by reference base, mutant base, SNP type, reference and alternate base coverage, and SnpEff annotations, including confidence, consequence, CDS position, CDS length and amino acid change (if applicable).

## 3 Discussion

This study introduces a comprehensive TILLING (Targeting Induced Local Lesions in Genomes) mutant resource for *B. napus* , offering a novel platform for gene discovery, functional genomics and targeted trait enhancement. Although TILLING has been successfully implemented in other crop species, its application in canola remains limited. This resource is ground‐breaking for several reasons, namely, it represents the first comprehensive spring canola TILLING resource, characterised for specific genes and made publicly available. Previously Gilchrist et al. (2013) developed a TILLING platform in DH12075 spring canola, but only characterised the mutant populations for a few hundred amplicons using a pooling strategy. Furthermore, this resource is based on a DH (doubled haploid) mutant platform, ensuring all mutations are fixed and homozygous. This feature offers a significant advantage for forward genetics, as the mutant seeds can be easily propagated, screened across multiple locations and exchanged among researchers. Lastly, the development of this resource includes a high‐quality genome assembly of the NRCDH4079 genotype, coupled with the identification of 1243 premature stop‐gain mutations. While this represents a substantial catalogue of high‐impact variants, these premature stop‐gain mutations occur in < 1% of the ~149 246 predicted genes in the *B. napus* genome, reflecting an inherent limitation of the current population size and mutation frequency. Nonetheless, this major advancement provides a powerful tool for both forward and reverse genetics, facilitating the identification of causal variants for key agronomic traits.

Regarding the mutational spectrum induced by EMS, we observed that 66.5% of SNPs are canonical GC → AT (C → T/G → A) transitions. While classical descriptions of EMS mutagenesis often emphasise that nearly all induced SNPs are GC → AT transitions, whole‐genome studies in several plant species demonstrate that substantial fractions of non‐canonical substitutions and small indels can also arise. For example, in rice, WGS of EMS‐mutant lines revealed ~70% of SNPs as GC → AT transitions, with the remainder representing other base substitutions (Yan et al. 2021). In *Cucurbita pepo* (squash), EMS‐induced mutations comprised 61%–80% GC → AT transitions, with non‐canonical transitions and transversions also observed (García et al. 2018). Similar observations have been reported in wheat and eggplant, where canonical transitions represented 37%–70% of total EMS‐induced mutations (Sidhu et al. 2015; Xiao et al. 2019). These findings suggest that the proportion of canonical EMS mutations is species‐ and context‐dependent, influenced by genomic sequence context, chromatin structure and the sensitivity of whole‐genome variant detection (Yan et al. 2021). In our study, variant calling applied stringent quality filters, read‐depth thresholds and controls for background variants, providing confidence that both canonical and non‐canonical SNPs represent genuine EMS‐induced mutations rather than false positives.

We demonstrated a key application of this TILLING resource for the improvement of seed quality traits, particularly meal protein content and fibre reduction, which are critical for expanding the value of canola beyond oil production. Using the TILLING population, we identified a specific variant associated with reduced meal ADF and increased meal protein levels, demonstrating the practical utility of this platform. Genetic mapping identified key genomic regions on chromosomes A1 and C1 linked to these traits, suggesting that these loci play a pivotal role in their coordinated regulation. This is further supported by the observed negative correlation between meal protein and fibre content, which highlights the need for targeted breeding strategies that optimise both attributes simultaneously.

This study reports, to our knowledge, the first identification of the A1/C1 chromosomal regions and their associated structural variants (SVs) linked to a compositional trait. Our results further demonstrate that this TILLING resource includes induced complex SVs—although large‐scale chromosomal variations are rare in EMS‐induced mutants, they are not entirely unprecedented in plants. EMS, a chemical mutagen, primarily induces point mutations and minor chromosomal alterations in plant genomes (Kim et al. 2006). Previous studies have indicated that EMS treatment may influence DNA methylation, potentially affecting genomic stability (Türkoğlu et al. 2023). In cowpea, higher concentrations of EMS have been associated with an increase in chromosomal abnormalities (Gnanamurthy and Dhanavel 2014), and EMS has been shown to cause partial deletions of chromosome 2D and translocations of 5AL/7BS in wheat (Wang, Guan, et al. 2024; Wu et al. 2024; Zhang, Wang, et al. 2024). However, significant chromosomal structural changes are typically rare in single EMS‐induced mutant events. Importantly, the structural variant identified in mutant DP125410314 did not originate from a high EMS dose; rather, it arose from the lowest concentration applied (0.1% EMS). This observation indicates that although higher EMS concentrations can increase the likelihood of chromosomal rearrangements, complex structural changes may still occur sporadically even at low treatment doses. Such rare events may reflect individual genome instability responses or interactions between EMS exposure and the doubled haploid (DH) production process. Given the low frequency of such structural variations induced by EMS, we suggest that the chromosomal translocation observed in mutant DP125410314 may have arisen either as a direct consequence of the EMS treatment or as an artefact of the doubled haploid (DH) protocol.

Beyond mutagenesis and variant discovery, this study also emphasises the role of structural variation in trait improvement (Hurgobin et al. 2018; Schilbert et al. 2023; Stein et al. 2017; Wang, van Dijk, et al. 2024; Zhang, Yang, et al. 2024). The discovery of a homoeologous non‐reciprocal translocation between A1 and C1 associated with reduced seed fibre content in DP125410314 underscores the influence of chromosomal rearrangements on phenotypic traits. The phenotypic differences observed in DP125410314 may be partially explained by the downregulation or loss of key genes located within the deleted A1‐1/A1‐2 region affected by the translocation. Specifically, the absence or strong downregulation of *MYB5*, *JAZ3* and *FYPP3*, all physically clustered in this region, points to these genes as potential contributors to the observed traits. *MYB5*, a transcription factor involved in seed coat development (Li, Milliken, et al. 2009; Xu et al. 2014), could play a key role in lignin deposition, and its loss may contribute to reduced ADF levels (Figure 7D). In support of this, downregulation of *MYB7/32/85*, transcription factors known to act downstream or in concert with *MYB5* in regulating phenylpropanoid biosynthesis (Xiao et al. 2021), was also observed. This suggests a coordinated disruption of the lignin biosynthesis regulatory network. On the other hand, JAZ3 and FYPP3 are implicated in modulating seed storage protein accumulation via the ABA signalling pathway, particularly through regulation of *ABSCISIC ACID INSENSITIVE 5 (ABI5)* (Dai et al. 2013; Ju et al. 2019). JAZ3 normally represses *ABI5* activity, and FYPP3 deactivates SnRK2, a kinase that activates ABI5. In the absence of both repressors, ABI5 is likely more active, leading to upregulation of downstream targets involved in seed maturation and protein storage (Ali et al. 2022; DeLisle and Crouch 1989; Zinsmeister et al. 2016) (Figure 7D). This is consistent with our observation of strong upregulation of *CRUCIFERIN 1/2/3* and *SEED STORAGE ALBUMIN 4* at embryo developmental stage E2, genes encoding major 12S and 2S storage proteins in Brassicaceae, respectively (Fujiwara et al. 2002). While structural variations introduce genetic diversity and create novel alleles beneficial for breeding, their incorporation requires careful evaluation to mitigate potential unintended consequences, such as linkage drag or the introduction of deleterious alleles. Nonetheless, leveraging structural variation in breeding programs holds immense potential for enhancing nutritional and industrial quality traits in canola.

The integration of haploid mutagenesis with genetic mapping represents a powerful strategy for prebreeding, enabling rapid identification of genomic regions associated with seed quality traits. The phenotypic analysis of the doubled haploid (DH) mutant population revealed substantial variation in key compositional traits, with differences of up to 7.2 percentage points (ppt) for seed oil, 6.9 ppt for seed protein, 8.0 ppt for meal protein and 5.8 ppt for meal ADF. These findings highlight the effectiveness of haploid mutagenesis in generating novel alleles with direct breeding applications.

## 4 Future Directions and Conclusion

The broader implications of this study have the potential to positively impact canola breeding. The TILLING platform not only addresses the historical bottleneck in genetic diversity caused by intense selection for high oil yield but also provides a sustainable approach to enhancing protein content and reducing fibre and antinutritional compounds. By integrating advanced genomic tools with phenotypic screening, breeders can efficiently identify and incorporate beneficial alleles into elite cultivars.

Targeted genome modifications using CRISPR and similar technologies offer a precise alternative to traditional mutagenesis methods such as EMS or radiation, which introduce widespread, random mutations. However, regulatory constraints have limited CRISPR's application primarily to gene discovery and functional validation rather than direct commercial crop development. Current policies vary across countries, creating significant barriers to commercialisation and international seed trade. Until global regulations align to support the deployment of gene‐edited crops, genome‐wide mutagenesis methods like EMS will continue to be essential for gene discovery and breeding applications.

In conclusion, this study establishes haploid mutagenesis and the TILLING platform as indispensable tools for *B. napus* genetic improvement. By providing a comprehensive genomic resource and demonstrating its practical utility in seed quality enhancement, this work paves the way for developing canola varieties that meet evolving nutritional and market demands. Furthermore, the findings have broader applications for other *Brassica* species, reinforcing the value of induced mutagenesis in crop improvement.

## 5 Materials and Methods

### 5.1 Plant Material and Growth Conditions

A homozygous doubled haploid line, NRCDH4079, derived from the Swedish *B. napus* cultivar, Topas, was used as the donor genotype for mutagenesis, as well as for genome and transcriptome sequencing. For DNA and RNA sample collection, canola plants were grown in standard 6‐in. pots (six pots per tray) filled with Pro‐Mix BX general‐purpose soil (75%–85% peat moss, perlite, vermiculite; medium drainage and water retention). Plants were maintained in a 765 ft^2^ controlled‐environment chamber at the LFK greenhouse, Innovation Place, Saskatoon. Day and night temperatures were set at 22°C and 20°C, respectively (with daytime temperatures typically running slightly warmer), and lighting was supplied by 400‐W high‐pressure sodium lamps under a 16‐h photoperiod. Plants were watered daily with municipal water supplemented with 20–20‐20 fertiliser (*N* = 100 ppm, *P* = 43 ppm, *K* = 83 ppm). To promote bolting, an additional 15–30‐15 fertiliser (*N* = 100 ppm, *P* = 128 ppm, *K* = 83 ppm) was applied to the chamber around week 3. Individual plants were covered with selfing bags one day before flowering using bamboo stakes, and plants were gently shaken each day after flowering to facilitate self‐pollination until pods began to form (approximately weeks 5–9). Plants were monitored daily for seed maturity, after which fertiliser was withheld to allow natural dry‐down. Mature plants were harvested and seeds were collected into labelled coin envelopes.

### 5.2 Microspore Mutagenesis and Creation of a Doubled Haploid Mutant Population

The *B. napus* microspore mutagenesis followed the method described by Ferrie et al. (2008). Briefly, donor plants were grown in environmentally controlled growth cabinets with a temperature of 20/15°C until buds were first observed, at which time the temperature was reduced to 10/5°C. Buds at the mid‐late uninucleate stage were selected, surface sterilised and macerated in half‐strength B5 medium (Gamborg et al. 1968) supplemented with 13% sucrose. The macerated material was filtered through a screen cloth into a sterile tube. The crude microspore suspension was centrifuged, the supernatant was decanted and the pellet was resuspended in B5‐13 medium. This washing process was repeated twice for a total of 3 centrifugation steps. After the final centrifugation step, the supernatant was decanted, and the pellet was resuspended in 10 mL of NLN‐13. The required amounts of EMS and microspore suspension were added to three tubes to result in EMS treatments of 0%, 0.1%, 0.2%, 0.3% and 0.4%.

The EMS‐treated microspores were incubated at room temperature for 1.5 h. Following incubation, the microspores were centrifuged, and the supernatant was discarded. The microspores were then washed three times with B5‐13 medium as described previously. The concentration of microspores in suspension was determined using a hemacytometer, and the required amount of NLN‐13 medium containing colchicine (10^−4^ M) was added to the tube to achieve a density of 10^5^ microspores/mL. Ten mL of microspore suspension was dispensed into each 100 × 15 mm sterile petri plate and placed in a 32°C incubator in the dark for 72 h, after which the colchicine was removed. The cultures were maintained at 24°C in the dark for the remainder of three weeks. Embryos that developed during this period were transferred to a shaker under light conditions to promote greening. For regeneration, the embryos were plated onto solid B5 media (1% agar, 1% sucrose, pH 5.8) and placed in the tissue culture room (22°C, 16 h photoperiod). After three weeks, the embryos had germinated and developed into plantlets. Plantlets with normal shoots were transferred to solid B5 medium with adjusted agar (0.8%) and sucrose (2%) concentrations for further development. Once a well‐developed root and shoot system was established, the plantlets were transferred to pots, grown to flowering and self‐pollinated, as described in the ‘Plant material and growth conditions’ section. Flow cytometry analysis was performed on the regenerated plantlets to determine their ploidy status. Based on these analyses, plantlets were classified as haploid, diploid/doubled haploid (DH) or mixoploid. Only lines confirmed as diploid/DH were advanced and evaluated for seed production. Seeds were harvested from each DH plant at maturity. The DH lines collectively comprised the mutant DH population.

### 5.3 Nuclear DNA Isolation for Genome Sequencing

A single plant of the *B. napus* genotype NRCDH4079 was grown as described in the ‘Plant material and growth conditions’ section. Fresh leaf tissue obtained from 3 to 4‐week‐old seedlings was homogenised in 200 mL of ice‐cold buffer composed of 0.01 M Trizma base, 0.08 M KCL, 0.01 M EDTA, 1 mM spermidine, 1 mM spermine, 0.5 M sucrose and 0.15% β‐mercaptoethanol, with a pH ranging from 9.4 to 9.5. The resulting homogenate underwent filtration using two layers of cheesecloth and one layer of miracloth. Nuclei were then collected via centrifugation at 800 rpm for 2 min to remove intact cells followed by 1800 *g* at 4°C for 20 min. The collected pellet was suspended in wash buffer (1× homogenisation buffer with the addition of 0.5% Triton‐X100) and subjected to centrifugation at 1800 *g* at 4°C for 15 min, repeated three times. Following the final wash, the nuclei were suspended in 10 mL of lysis buffer comprising 100 mM TrisCl, 100 mM NaCl, 50 mM EDTA and 2% SDS. Genomic DNA of high molecular weight was extracted through traditional proteinase K digestion (0.05 mg m L‐1; at 65°C for 2 h), followed by phenol/chloroform extraction, isopropanol precipitation overnight, 70% ethanol wash and RNAase A treatment. This was followed by another round of phenol/chloroform extraction, chloroform extraction and ethanol precipitation. The quantification of genomic DNA was carried out using the PicoGreen dsDNA kit from Molecular Probes.

### 5.4 Oxford Nanopore DNA Library Preparation and Sequencing

High‐molecular‐weight DNA was size‐selected using the Short‐Read Eliminator kit (SKU 102–208‐300; Circulomics/PacBio). One μg of size‐selected DNA was then prepared for library construction using the 1D genomic DNA by ligation method for Oxford Nanopore sequencing on the MinION (SQK‐LSK109; Oxford Nanopore Technologies). Genomic DNA libraries were prepared and sequenced following the nanopore protocol (https://nanoporetech.com/documentation/results?category=prepare&topic=library‐prep‐protocols). The sequencing run was programmed to last 72 h, with data storage parameters configured accordingly.

### 5.5 Genome Assembly

Basecalling was carried out using Guppy version 3.6.0, and reads were filtered using NanoFilt (v2.8.0) (De Coster et al. 2018) for a minimum length of 8000 bases and a minimum quality score of 8. The initial assembly was completed using SMARTdenovo (v1.4.13) with default parameters (Liu et al. 2021). Racon (*v*, *m* = 8, *x* = −6, *g* = 8) (Vaser et al. 2017) was used to polish the draft assembly with the same long‐read data that were used to generate the assembly. Medaka (v1.2.3, https://github.com/nanoporetech/medaka) was used to create consensus calls from the polished draft assembly using the r941_min_high_g360 model and default parameters. The Medaka output was then polished a second time with Racon using short‐read Illumina data and default parameters to improve the contig sequence quality. BUSCO (v5.7.0) (Simão et al. 2015) scores were calculated at each stage in the genome assembly development—draft, Racon long‐read, Medaka, Racon short‐read and finally pseudomolecule—and were run using three lineages—embryophyta_odb10, eudicots_odb10 and brassicales_odb10.

### 5.6 Bionano Optical Mapping for Scaffolding

Genome optical mapping was performed using the Direct Label and Stain (DLS) chemistry in a Bionano Saphyr platform (Bionano Genomics, San Diego, California). Ultra‐high‐molecular‐weight nuclear DNA (uHMW nDNA) was isolated from approximately 0.5 g of fresh young leaf tissue from a 3–4‐week‐old plant grown (as described in the ‘Plant material and growth conditions’ section) from seeds collected from the same plant used for gDNA extraction for Nanopore sequencing. DNA labeling was performed as per manufacturer's protocols, with some modifications (https://Bionanogenomics.com/) (Rabanal et al. 2022; Wang et al. 2021) using a centrifugation speed of 2500 x g. Approximately 800 ng of uHMW DNA was labelled using the DLS approach and the resulting sample loaded into a Saphyr G2.3 chip and molecules separated using a Saphyr Analyser. The processed collected DLE‐1–labelled molecule dataset was filtered to create a subset of 445 513 molecules with a minimum size of 450 kbp and molecule N50 length of 603.428 Kbp. Filtered molecules were then assembled on the Bionano Access platform using the Tools version 1.6.1 software, configured with the ‘non‐haplotype, no extend and split, no cut CMPR’ settings. The final assembly had 200× effective molecule coverage, resulting in a total assembly length of 991.61 Mbp across 54 maps, with map N50 length of 28.52 Mbp and maximum map length of 58.54 Mbp.

### 5.7 Hi‐C Validation

Hi‐C data was generated using the Arima‐HiC kit (Catalogue #A510008) according to the manufacturer's protocol (https://arimagenomics.com/wp‐content/files/User‐Guide‐Arima‐HiC‐for‐Plant‐Tissues.pdf). The library was quantified using the dsDNA High Sensitivity (HS) assay on a Qubit 4 Fluorometer (ThermoFisher, USA), and the average fragment length was assessed using the D1000 assay on a TapeStation 4200 system (Agilent, USA). Paired‐end 150 bp reads were generated using the Illumina NovaSeq 6000 platform. For validation of the genome assembly, the Hi‐C contact map was generated using the 3D‐DNA (v180922) script and visualised using Juicebox (v1.11.08).

### 5.8 Repeat Annotation

Whole‐genome repeat annotation was performed using RepeatMasker v4.1.5 (http://www.repeatmasker.org) with the parameters ‘‐no_is ‐nolow.’ This analysis utilised the *Brassica* Transposable Element (TE) library, which was manually curated as part of the Canadian Canola pan‐genome sequencing project. Additionally, the EDTA (v2.2.0) pipeline (Ou et al. 2019) was employed for the comprehensive annotation of full‐length Long Terminal Repeats (LTRs) and their age distribution. The identified full‐length LTRs were further categorised into families using the TEsorter (Zhang et al. 2022). The characterisation of putative centromere regions for each chromosome in the genome was performed based on the distribution patterns of the Centromeric retrotransposon of *Brassica* (CRB) and the Centromeric tandem repeats of *Brassica* (CentB) repeats (Koo et al. 2011; Lim et al. 2007). MITEtracker (Crescente et al. 2018) was utilised to identify potential Miniature Inverted‐repeat Transposable Element (MITE) families within the genome. These candidates were then classified based on their terminal inverted repeat (TIR) structure. The genomic distribution of MITEs was investigated using the bedtools toolkit (Quinlan 2014).

### 5.9 Developmental Transcriptome Analysis

Plants were grown from seeds collected from the same plant used for gDNA extraction for Nanopore sequencing, as described in the ‘Plant material and growth conditions’ section. Eighteen distinct tissue samples were collected at various developmental stages throughout the plant's life cycle (Table S6). These included sprouts (4 days after germination, DAG), cotyledons (8 DAG), second leaves (13 DAG), fourth leaves (17 DAG), mature leaves (34 DAG) and senescing leaves (85 DAG), as well as roots (17 DAG), inflorescence (51 DAG) and flowers (52 DAG). Additionally, seeds were sampled at eight developmental stages between 4 and 36 days post‐anthesis (DPA), at 4‐day intervals. For each tissue type, a minimum of three independent biological replicates was analysed. Twelve plants were grown for tissue collection. Root tissues were harvested from three of these plants (one plant per biological replicate). From the remaining six plants, three were used to collect leaf and flower tissues, while all six plants contributed to tissue sampling at different seed developmental stages (two plants per biological replicate). For cotyledon collection, two seeds were sown per replicate, and for sprout sampling, six seeds were used per replicate. Biological replicates were processed separately, and RNA was extracted individually for each replicate.

Total RNA from all tissues was isolated using the RNeasy Plant Mini Kit (Qiagen, http://www.qiagen.com/) according to the manufacturer's instructions. Illumina TruSeq RNA sequencing libraries were constructed following the standard preparation guide (Illumina, http://www.illumina.com/) and sequenced (paired‐end, 125 cycles) using the Illumina platform to produce approximately 13 million reads per replicate. Before gene annotation using Breaker2 pipeline, short Illumina RNA‐seq reads were filtered using Trimmomatic (v0.39) (Bolger et al. 2014) with default settings by trimming adapter and low‐quality sequences and removing reads shorter than 75 bp.

### 5.10 Gene Annotation

Gene annotation was performed on a soft‐masked version of the NRCDH4079 assembly. To generate the repeat‐masked genome, we first constructed a comprehensive and non‐redundant *Brassica* ABC repeat library by characterising transposable elements (TEs) de novo in representative genomes of all three diploid *Brassica* lineages, *B. rapa* (A genome), *B. nigra* (B genome) and *B. oleracea* (C genome), as well as the amphidiploid *B. napus* (AC genome). For each genome, TEs were identified using EDTA with default structural and homology‐based detection parameters, generating curated sets of LTR retrotransposons, DNA transposons (TIR and non‐TIR), Helitrons and other repeat subclasses. The four TE sets were then merged using the PanEDTA pipeline (https://github.com/oushujun/EDTA), which performs clustering, redundancy filtering and classification to produce a unified *Brassica* ABC repeat library. Because EDTA does not effectively capture tandem repeats, we manually added centromeric satellite repeats (CentB) and 5S and 45S rDNA arrays from published *Brassica* references (Chen et al. 2025; Lim et al. 2005). The resulting *Brassica* ABC library, comprising ~19 000 classified elements, was used for genome‐wide soft masking with RepeatMasker (v4.2.1) (Chen 2004), generating the repeat‐masked genome used as input for gene prediction.

Protein‐coding genes were annotated using the Braker2 pipeline (Brůna et al. 2021), which integrates both ab initio predictions and evidence‐based methods. Protein datasets from *B. rapa, B. oleracea* and *B. napus* reference genome annotations (He et al. 2021) along with RNA‐seq data from 18 tissues (Table S6) were provided to Braker2 (Brůna et al. 2021; Stanke et al. 2008; Stanke et al. 2006) to facilitate annotation of protein‐coding genes on the soft‐masked genome. To refine and enrich the annotations, PASA (v2.5.3) was used to assemble transcripts and incorporate transcript alignment evidence into the Braker‐generated gene models. Functional annotation of the predicted genes was carried out using BLAST+ and InterProScan by aligning gene models to homologous proteins and protein domains in the UniProt and Pfam databases. This comprehensive annotation process resulted in the identification of 149 246 genes in the genome.

### 5.11 Synteny Analysis

Synteny analysis was performed as described previously (Kagale et al. 2014). Briefly, sequence similarity was identified through BLASTP analysis, comparing the predicted proteins with the *A. thaliana* proteome (TAIR10 release). Hits with an *E*‐value ≤ 1e−20, within the top 40% drop from the highest bit score, were selected for further investigation. Syntenic chains between *B. napus* and *A. thaliana* gene pairs were determined using DAGChainer (Haas et al. 2004) with default settings. In cases where a *B. napus* gene appeared in multiple syntenic chains due to duplication in the *A. thaliana* genome, the pair with the lower scoring chain was excluded from analysis. The resulting syntelog table was created by mapping the syntenic chains onto the chromosomes of *B. napus* .

### 5.12 Functional Gene Annotation: Gene Ontology, KEGG and TF Families

GOMAP‐singularity version 1.3.8 was used for gene ontology (GO) annotation of the assembled NRCDH4079 genome (Wimalanathan and Lawrence‐Dill 2021). R package GOfuncR version 1.18.0 (Grote 2018) was used to retrieve GO term descriptions and root categories. GO terms were manually inspected to remove non‐plant GO terms. GhostKOALA was used for KEGG (Kyoto Encyclopedia of Genes and Genomes) orthology assignments (Kanehisa et al. 2016) and then KEGG Mapper was used to reconstruct the KEGG pathway (Kanehisa et al. 2022). Mercator4 version 5.0 was used to identify transcription factors (TFs) from the NRCDH4079 genome (Schwacke et al. 2019).

### 5.13 PanKmer Analysis

To investigate the genetic relationships and shared genomic features between different *B. napus* genome assemblies, we employed the PanKmer pipeline (Aylward et al. 2023), a k‐mer‐based comparative genomics approach designed for identifying shared and unique sequences across multiple genomes.

### 5.14 Validation of the Exome Array

To evaluate capture efficiency of the *B. napus* exome array and determine optimal multiplexing levels, exome sequencing was performed on 24 *B. napus* lines, including 20 mutants, three cultivars (DH12075, NRCDH4079, Express617) and a proprietary control line with a known mutation. Samples were sequenced in 12‐ and 24‐sample multiplexed runs on an Illumina HiSeq, generating ~15.7 million (6× coverage) and ~8.9 million reads (3× coverage) per sample, respectively (Table S19). Over 99% of captured reads aligned from all three cultivars to the reference genome, with uniform coverage across all 19 chromosomes (Figure S11A). However, 16‐sample multiplexing outperformed 28‐sample multiplexing, achieving 100% gene coverage for > 30 000 genes and 60% coverage for most remaining genes (Figure S11B). In contrast, 24‐sample multiplexing resulted in 30%–70% coverage for most genes (Figure S11C). The known mutation in the control line was successfully detected under both conditions. These results confirm that the array efficiently captures a substantial portion of the gene space, with deeper sequencing enhancing performance. Consequently, we transitioned to NovaSeq for higher‐throughput mutant genotyping, achieving ~30× coverage across all DH lines.

### 5.15 DNA Isolation for Exome Sequencing of Mutant Lines

Leaf tissue from NRCDH4079 mutant seedlings was collected at the 4‐ to 12‐leaf stages, approximately three to four weeks after sowing or transplanting. Using a 5 mm punch sampler, 16 leaf discs were taken from a single leaf, with the sampler cleaned between samples using 2% sodium hypochlorite solution to prevent contamination. The discs (approximately 80 mg fresh weight) were placed in collection microtubes, kept on ice and subsequently stored at −80°C. Following freeze‐drying for 48 h and disruption with a TissueLyser, genomic DNA was extracted using the AutoGen Prep 965 plant protocol. The DNA was resuspended in 50 μL of ultrapure water and stored at −20°C. DNA concentrations were quantified using the Quant‐iT dsDNA Broad‐Range Assay kit.

### 5.16 Nextera Flex Library Preparation and Sequencing

To individually label the gDNA from each line with a unique pair of index adaptors, the first four steps of the Nextera Flex for Enrichment protocol (Illumina: Tagment Genomic DNA, Post‐Tagmentation Clean Up, Amplify Tagmented DNA and Clean Up Libraries) were performed with slight modifications. We used 100 ng of gDNA per line in 10 μL volume and processed libraries in batches of 48 to minimise errors. Reagent volumes were reduced to one‐third of the recommended amounts, and precision pipette tips were used. Incubation and PCR conditions remained unchanged. Samples and reagents were mixed on a high‐speed microplate shaker when recommended. The first 96 preparations used Nextera Unique Dual Indices; subsequent plates used IDT for Illumina NXT 10 nt indices, totaling 1 UD plate and 12 NXT plates for 1240 libraries. The eBLT PCR program had nine cycles as suggested. For the final Clean Up Libraries step, NucleoMag beads replaced AMPure XP beads, and ultrapure nuclease‐free water was used for elution. Initial quality checks of libraries were performed with an Agilent Bioanalyzer (DNA 1000 chip, Agilent Technologies), and concentrations were determined using the Quant‐iT dsDNA Broad‐Range Assay kit (ThermoFisher) following manufacturers' instructions. The library preparation was repeated for gDNA samples where the library concentration was below 8 ng/μL. Libraries were either pooled immediately or frozen at −20°C.

### 5.17 Pooling Nextera Libraries

Sample libraries were pooled equally by mass, creating pools of 12 samples each with a final concentration of 1.1 μg (91.67 ng per library). This plexity provided better sequencing depth than 24‐sample pools. Lower plexity was not feasible due to reduced reaction volumes. From each pool, 100 ng was reserved for future exome capture enrichment testing via real‐time PCR and potential targeted sequencing. Libraries were either pooled immediately or stored at −20°C for up to 80 days before exome capture, with most captured within 30 days.

### 5.18 Exome Capture and Illumina Sequencing

Exome capture was performed following the SeqCap EZ HyperCap workflow User's Guide (Roche, Chapter 5) with modifications, including vacuum centrifugation. Eight exome capture reactions were performed per batch, covering 96 libraries. The SeqCap EZ Prime Developer Probe was aliquoted into 8‐pool aliquots of 18 μL (2.25 μL per pool, half the recommended volume).

To initiate exome capture, 1 μg of pooled library sample was combined with 2 μL of IDT Illumina Xgen Universal Blockers and 10 μL of SeqCapEZ Developer Reagent (Roche) in a 1.5 mL microfuge tube. The IDT blockers replaced HyperCap Universal Blocking Oligos, as libraries were created using Illumina indices. Since the genomic DNA was from ‘canola’, SeqCapEZ Developer Reagent replaced COT Human DNA. The tube was pierced with a 20‐gauge needle and dried down in a DNA vacufuge (Eppendorf) set to aqueous mode at 60°C. During this process, 1X wash buffer solutions were prepared from the SeqCap EZ Hybridisation and Wash Kit (Roche) and stored at room temperature. Once dry, tubes were sealed with 1 cm dot stickers. To each tube, 7.5 μL of Hybridisation Buffer and 3 μL of Hybridisation Component A were added, followed by vortexing for 10 s to resuspend. Tubes were incubated at 95°C for 10 min for denaturation. Meanwhile, 2.25 μL of SeqCap EZ Prime Developer Probe was aliquoted into eight 0.2 mL PCR tubes. An additional 18 μL of nuclease‐free water was added to the probe aliquot, mixed, and 2.25 μL was transferred to each PCR tube, yielding half the recommended probe amount (4.5 μL total). The denatured sample (~10.5 μL) was spun down and added to the PCR tubes with the diluted probe. Tubes were then placed in a thermocycler for hybridisation following the manufacturer's recommended protocol.

Before washing, capture beads were equilibrated at room temperature. The diluted 1X stringent wash buffer and one volume of diluted 1× wash buffer I were preheated to 47°C in a Thermostat incubator (Eppendorf) for 2 h. After 1 h of preheating, the capture beads were washed according to the protocol. After washing, 50 μL aliquots of beads were transferred into eight new 0.2 mL PCR tubes. The corresponding hybridisation reaction was added to each PCR tube, mixed by vortexing, briefly spun down and incubated at 47°C for 45 min. Tubes were gently flicked to mix every 15 min.

After incubation, bead‐bound DNA was washed as follows: First, wash buffer I and two stringent buffer washes were performed at 47°C in a Thermostat incubator. Next, second wash buffer I, wash buffer II and wash buffer III were applied at room temperature in the same microcentrifuge tubes. After washing, 15 μL of PCR‐grade water (Roche SeqCap EZ Accessories Kit v2) was added to each sample. The SeqCap EZ Accessories Kit was used in place of the HyperCap Target Enrichment Kit to reduce costs. The entire bead and water mix was used as the template for Ligation‐Mediated (LM) PCR. LM‐PCR was performed as per the manufacturer's specifications. The amplified captured DNA was purified using an equal volume (50 μL) of AMPure Beads (Machery‐Nagel), following the described protocol. Library concentrations were quantified using the Quant‐iT dsDNA Broad‐Range Assay kit (ThermoFisher), according to the manufacturer's instructions. Fragment sizes ranged from approximately 300 bp to 500 bp. The purified captured library pools were stored at −20°C for up to one month prior to sequencing. Sequencing of 1240 successfully prepared libraries was carried out on four NovaSeq 6000 flow cells, generating a total of 11.83 Tb of data.

### 5.19 Exome Analysis

Raw reads were trimmed with trimmomatic (v0.33) (Bolger et al. 2014) and quality assurance was completed with fastqc (v0.12) on the raw as well as the trimmed reads. Trimmed reads were aligned to the NRCDH4079 reference genome using Bowtie2 (v2.4.3) (Langmead and Salzberg 2012). Alignment statistics were calculated with SAMtools (v1.21.1) (Li, Handsaker, et al. 2009). Variants were identified using BCFtools (v1.15) mpileup and called with BCFtools call. Resulting per‐sample variant files were filtered with following parameters: quality score ≥ 30, minimum depth of 5, maximum depth of 250 filtered using VCFtools (v0.1.13) (Danecek et al. 2011). These settings were chosen based on iterative comparisons to balance sensitivity and specificity in calling high‐quality EMS‐induced variants. Variant files were split into SNP‐only and indel‐only datasets using BCFtools view. Indels underwent additional filtering with stricter thresholds: quality ≥ 50 and minimum read depth ≥ 10. Variants shared by five or more independently mutagenised lines were removed to reduce false positives, since it is highly unlikely that EMS would induce the same mutation independently in multiple lines.

### 5.20 RNA Sequencing of Embryonic and Seed Coat Development

Plant lines derived from the DP125410314 mutant and NRCDH4079 were grown under controlled environmental conditions, with a 16‐h light and 8‐h dark photoperiod. Daytime and nighttime temperatures were maintained at 22°C and 20°C, respectively, with light intensity kept between 120 and 150 μmol m^−2^ s^−1^. Embryo and seed coat tissues were dissected following established methods (Gao et al. 2022), using precision tools including a dissecting microscope, fine‐tipped forceps (Dumont 55, catalogue #11295‐55; Fine Science Tools, Foster City, CA, USA) and dissecting needles (catalogue #10130‐05; Fine Science Tools). Dissections were performed in a solution containing 4.8% sucrose and 0.1% RNALater stabilisation reagent (catalogue #AM7021; Ambion, TX, USA). RNA sequencing was carried out on seed compartment tissues collected at four developmental stages, including E4 to E7 (embryo) and S4 to S7 (seed coat), corresponding to 14, 18, 22, and 26 days post‐anthesis.

Total RNA was extracted from each stage embryo and seed coat sample following the protocol of RNAqueous micro kit (Ambion, catalogue no. 1927). RNA‐seq libraries were prepared using the NEBNext Single Cell/Low Input RNA library Prep Kit for Illumina (New England Biolabs, Cat# E6420L) with the following modifications. For inputs of RNA with 2000 or more cells/embryo, 10 PCR cycles for cDNA amplification were used. For inputs of RNA with less than 2000 cells/embryo, 13 PCR cycles were used. 20 ng of purified cDNA was used as input for the fragmentation/end repair step. For PCR enrichment of the adaptor‐ligated DNA, the index primers were used at 1/5 provided concentration and 8 PCR cycles were completed. The quality of each library was checked on a DNA1000 chip on the 2100 Bioanalyzer (Agilent Technologies Inc.) and the concentration was determined by qPCR using the KAPA SYBR FAST ABI Prism qPCR Kit (Kapa Biosystems) and the StepOnePlus Real‐Time PCR System (Applied Biosystems). Equimolar concentrations of the libraries were pooled and a concentration of 18 pM + 5% PhiX (12.5 pM) was used for clustering per lane of a flow cell on the cBOT (Illumina). The samples were sequenced (2 × 150 cycles, paired‐end reads) on the HiSeq2500 platform.

RNA‐seq reads were first reprocessed by trimming the adaptor sequences, filtering low‐quality reads and eliminating short reads using Trimmomatic (Bolger et al. 2014), with the argument ILLUMINACLIP:TruSeq3‐SE:2:30:10 SLIDINGWINDOW:5:20 MINLEN:75. Filtered reads were aligned to the DH4079 genome using STAR (v2.7.5a) (Dobin et al. 2013). Transcript abundance was estimated using the RSEM (v1.3.3) algorithm (Li and Dewey 2011). Differential expression analysis was performed with DESeq2 (Love et al. 2014) in R, using raw counts as input. Differentially expressed genes were identified based on an adjusted *p*‐value < 0.05 (Benjamini–Hochberg correction) and absolute log2 fold‐change ≥ 1. Principal component analysis (PCA) and sample clustering were performed to assess overall data quality and detect batch effects.

### 5.21 Visualisation of Large Structural Variants (LSV‐Viz)

Using the BAM files generated using bwa (v0.7.18), bedtools coverage (v2.31.1) was applied to determine the gene coverage for all samples. Gene counts for each sample were compiled into a data matrix, TPM normalised per sample and then used as input for LSV‐viz. LSV‐viz was developed in Python using NumPy, Pandas, and matplotlib modules and Python 3. In the absence of a reference sample, the sample median per gene served as the denominator when calculating the gene coverage ratio for all samples. These ratios were visualised using a sliding window average of 20 and a step size of 5. LSVs were then identified, and their corresponding gene and genome coordinates were extracted for further analysis.

### 5.22 Field Phenotyping of the Mutant Population and the Mapping Population Derived From DP125410314 × NRCDH4079

#### 5.22.1 Mutant Population

In 2020, 1200 double haploid lines from the mutant population produced via EMS microspore mutagenesis were grown at two locations: Carman, Manitoba (49.49077, −97.99674), and Saskatoon, Saskatchewan (52.19221, −106.5127). All lines were planted in 2‐m rows with 0.5‐m spacing between rows using a non‐randomised experimental block design. Five inbreds were used as diagonal checks in the experiment to allow calculation of row and column adjustments and provide an estimate of residual variance (Gilmour et al. 1997). A Corteva elite inbred (CHK) and the original DH donor, NRCDH4079, were included as entries in the field experiment. Two replicate rows were used and evaluated across both sites. At maturity, seed samples were hand‐harvested from the center 1‐m of the row for each entry. Samples were scanned with a NIRSystem 6500 (Foss, Hillerod, Denmark), which was calibrated to estimate seed oil, seed and meal protein, and meal acid detergent fibre (ADF). The moisture of each sample was also estimated and used to adjust all compositional data to a dry basis.

Statistical analysis of the phenotypic data was performed using ASReml software (Butler et al. 2017) to estimate best linear unbiased estimator (BLUE) values for all traits on all of the entries. Data was analysed first using a random model to estimate variance components:

$$Y=m+L+G+G^{*}L+\mathrm{at}\left(L\right)\cdot \left(\mathrm{Row}\;\text{Column}\right)+\mathrm{at}\left(L\right)\cdot \text{Residual},$$

where *m*, overall mean of dataset = fixed effect; *L*, location = fixed effect; *G*, genotype = random effect; G*L = location × genotype interaction = random effect; at(*L*)·(Row Col) = row & column effect for each individual in each location = random effect; as(*L*)·Residual = random effect with an AR1 × AR1 structure.

Data was then analysed using a fixed model to obtain the BLUE values:

$$Y=m+L+G+G^{*}L+\mathrm{at}\left(L\right)\cdot \left(\mathrm{Row}\;\text{Column}\right)+\mathrm{at}\left(L\right)\cdot \text{Residual},$$

where *m*, overall mean of dataset = fixed effect; *L*, location = fixed effect; *G*, genotype = fixed effect; G*L, location × genotype interaction = fixed effect; at(*L*)·(Row Col) = row & column effect for each individual in each location = random effect; at(*L*)·Residual, random effect with an AR1 × AR1 structure.

One line (DP125410314) was selected from the population for very low meal ADF compared to the NRCDH4079 donor line, for use as a mutant parent to create a population to map the genetic basis of the low ADF phenotype.

#### 5.22.2 Mapping Population

*F*_1_ seed was made by crossing DP125410314 with the original donor, NRCDH4079. The *F*_1_ seed was harvested, then planted, grown to flowering and self‐pollinated to produce *F*_2_ seed. *F*_2_ seed was planted, and 200 *F*_2_ plants were grown out in the greenhouse and self‐pollinated. Tissue was sampled from each *F*_2_ plant for DNA extraction for genotyping. Mature *F*_3_ seed was harvested individually from each *F*_2_ plant to produce a mapping population of *F*_3_ lines. One hundred and eighty‐four (184) of the *F*_3_ lines plus the parents of the original cross (NRCDH4079, DP125410314) were planted in a nursery experiment in 2022, at the same two locations where the original mutant population had been evaluated in 2020. Several replicated checks were included to provide an estimate of residual. At maturity, seed samples were harvested from all entries. All samples were scanned on the same NIRS instrument as was used for the 2020 experiment, and data on the same four compositional traits (seed oil, seed protein, meal protein, meal ADF) was captured and adjusted to dry basis prior to statistical analysis. The same software and approach were used in the analysis of the mapping population as for the mutant population, except that there were no row and column adjustments involved. Data was analysed first using a random‐effects model to obtain estimates of variance components:

$$Y=m+L+G+G^{*}L+\text{Residual},$$

where *m*, overall mean = fixed; *L*, location effect = fixed; *G*, genotype effect = random; G*L, genotype × location effect = random; Residual, random.

Data was then analysed using a fixed‐effect model to obtain BLUE values:

$$Y=m+L+G+G^{*}L+\text{Residual},$$

where *m*, overall mean = fixed; *L*, location effect = fixed; *G*, genotype effect = fixed; G*L, genotype × location effect = fixed; Residual, random.

### 5.23 MutMap Strategy

Lines from the mapping population were ranked based on phenotype (seed protein, meal protein and ADF content), from lowest to highest. The 25 samples with the highest phenotypic values and the 25 samples with the lowest values were combined after read trimming to form pseudo ‘mutant’ and ‘reference’ population bulks. For traits seed protein and meal protein, the highest 25 samples represented the pseudo‐mutant population, while the lowest 25 represented the reference population. For fibre (ADF), the assignments were reversed. The sequence data from combined pseudo‐bulks were aligned to the reference genome using Bowtie2 (Langmead and Salzberg 2012). SNPs were called using BCFtools mpileup and filtered with the following parameters: minimum quality score of 30, minimum depth of 10 (using BCFtools filter). Filtered SNPs were analysed using a MutMap strategy (Abe et al. 2012), outlined below.

Pseudo bulk samples representing the reference population and the mutant population were compared to each other. For each SNP in the mutant or reference population, at any given locus the proportion of bases that matched the reference base versus the alternate was calculated. This ratio, defined as: MutMap ratio = (mutant bases)/(total bases mapped to a given locus), provides insight into the prevalence of a mutation within the population. SNPs with higher ratios (e.g., ≥ 0.9) are considered of interest, particularly when multiple adjacent loci exhibit high MutMap ratios. The same calculations were carried out on the pseudo‐reference bulk to confirm that the same loci are not undergoing mutations at comparable rates in the reference population.

## Author Contributions

S.K., J.S.S.A. and D.C. conceived and designed the study. A.T.T., M.K., T.M. and Y.B. prepared the sequencing libraries and carried out Oxford Nanopore Technologies (ONT) sequencing. Genome assembly and repeat and protein‐coding gene annotation were performed by M.W.K., K.F., V.L., K.C.K., H.Y. and S.P. A.M.R.F. conducted mutagenesis and developed the doubled haploid population. Variant analysis was carried out by M.W.K., D.C. and S.K. I.A.P.P. and A.G.S. designed the Exome Capture arrays, which were validated by Y.T. and S.K. Field experiments, data analysis and genetic mapping were conducted by S.S., U.A., F.T., I.F., C.K., S.G., D.C. and J.S.S.A. C.M., V.B. and C.G. handled data visualisation and web design. J.B.N. and M.A.S. performed the functional characterisation of A1/C1 structural variant. The manuscript was written by S.K., M.W.K. and J.S.S.A., with all authors contributing to the final version.

## Funding

This work was supported by the National Research Council Canada, Pioneer Hi‐Bred Production Company and The Agriculture Development Fund from the Government of Saskatchewan.

## Conflicts of Interest

The authors declare no conflicts of interest.

## Supporting information


**Supporting Information: 1.** A syntelog matrix representing individual *Arabidopsis thaliana* genes and the corresponding *Brassica napus* homeologues.

**Figure S1:** Overview of the haploid microspore mutagenesis protocol.
**Figure S2:** Histogram showing the number of mutant lines recovered at each EMS concentration (0.0%, 0.1%, 0.2%, 0.3%, 0.4%).
**Figure S3:** Phenotypes of interest observed in EMS mutagenized *B. napus* lines.
**Figure S4:** Distribution of days to flowering phenotype across EMS concentrations in mutagenized population.
**Figure S5:** Transcription factors (TF) families in the *B. napus* genotype DH4079 genome.
**Figure S6:** Distribution of full length LTRs in the *B. napus* genotype DH4079 genome.
**Figure S7:** Age distribution of full length LTRs.
**Figure S8:** Distribution of Miniature Inverted repeat transposable elements (MITEs).
**Figure S9:** Distribution of SNPs across EMS concentrations in mutagenized population.
**Figure S10:** Distribution of InDels across EMS concentrations in mutagenized population.
**Figure S11:** Validation of the exome capture array.

**Table S1:** EMS concentration (%) applied during generation of the mutagenized lines.
**Table S2:** Days to Flowering data for mutant lines.
**Table S3:** Summary of Oxford Nanopore and Illumina data generated for *B. napus* genotype DH4079 genome assembly.
**Table S4:** *B. napus* genotype DH4079 genome assembly statistics.
**Table S5:** BioNano optical map‐based scaffolding.
**Table S6:** Summary statistics for the developmental transcriptome.
**Table S7:** Repeat composition in *B. napus* genotype DH4079 genome.
**Table S8:** Tentative centromere locations *B. napus* genotype DH4079 chromosomes.
**Table S9:** Summary of Exome Capture coverage in the *B. napus* genotype DH12075 genome.
**Table S10:** List of DH4079 genes missing from the DH12075 exome capture array.
**Table S11:** Summary of sequencing data from Exome Captures of 1239 EMS‐mutagenized lines.
**Table S12:** Summary of variants in the DH4079 mutant lines.
**Table S13:** Distribution of SNPs across the AA and CC sub‐genomes.
**Table S14:** Phenotypic data (BLUEs) for mutant lines having mutations in the acyl‐lipid pathway genes.
**Table S15:** Raw NIRS phenotype data for mutant population.
**Table S16:** BLUEs phenotype data for mutant population.
**Table S17:** Raw NIRS phenotype data for mapping population.
**Table S18:** BLUEs phenotype data for mapping population.
**Table S19:** Validation of exome capture array—sequencing coverage at 16‐plex and 28‐plex exome capture assays.

## Data Availability

The genome assembly and annotation files are available at Zenodo (https://doi.org/10.5281/zenodo.15627256). Raw sequencing data have been deposited in the National Center for Biotechnology Information (NCBI) under the BioProject accession PRJNA1273955 (http://www.ncbi.nlm.nih.gov/bioproject/1273955). The Bio sample IDs for raw sequencing data are as follows: SAMN48970102 ( *B. napus* NRCDH4079 RNA‐seq seed coat and embryo), SAMN48970101 ( *B. napus* NRCDH4079 RNA‐seq developmental stages), SAMN48970100 ( *B. napus* NRCDH4079 exome capture sequencing), SAMN54468406 (*B. napus* NRCDH4079*DP125410314 mapping population sequencing), SAMN48969851 ( *B. napus* NRCDH4079 HiC sequencing), SAMN48969850 ( *B. napus* NRCDH4079 short‐read sequencing) and SAMN48969849 ( *B. napus* NRCDH4079 Oxford Nanopore Sequencing). Code Availability: No new code was generated for this project.
