# Supplementary material for: Haploid Mutation Mapping Identifies a Homoeologous Non‐Reciprocal Translocation Linked to Reduced Fibre and Enhanced Protein in Brassica napus
Source: Plant Biotechnol J. 2026 Jan 29;24(5):3219–43. doi: 10.1111/pbi.70535 (PMC13110153; doi:10.1111/pbi.70535)
Supplement: Supplementary file 2 — Figure S1: Overview of the haploid microspore mutagenesis protocol. Figure S2: Histogram showing the number of mutant lines recovered at each EMS concentration (0.0%, 0.1%, 0.2%, 0.3%, 0.4%). Figure S3: Phenotypes of interest observed in EMS mutagenized B. napus lines. Figure S4: Distribution of days to flowering phenotype across EMS concentrations in mutagenized population. Figure S5: Transcription factors (TF) families in the B. napus genotype DH4079 genome. Figure S6: Distribution of full length LTRs in the B. napus genotype DH4079 genome. Figure S7: Age distribution of full length LTRs. Figure S8: Distribution of Miniature Inverted repeat transposable elements (MITEs). Figure S9: Distribution of SNPs across EMS concentrations in mutagenized population. Figure S10: Distribution of InDels across EMS concentrations in mutagenized population. Figure S11: Validation of the exome capture array. [file PBI-24-3219-s001.pdf]

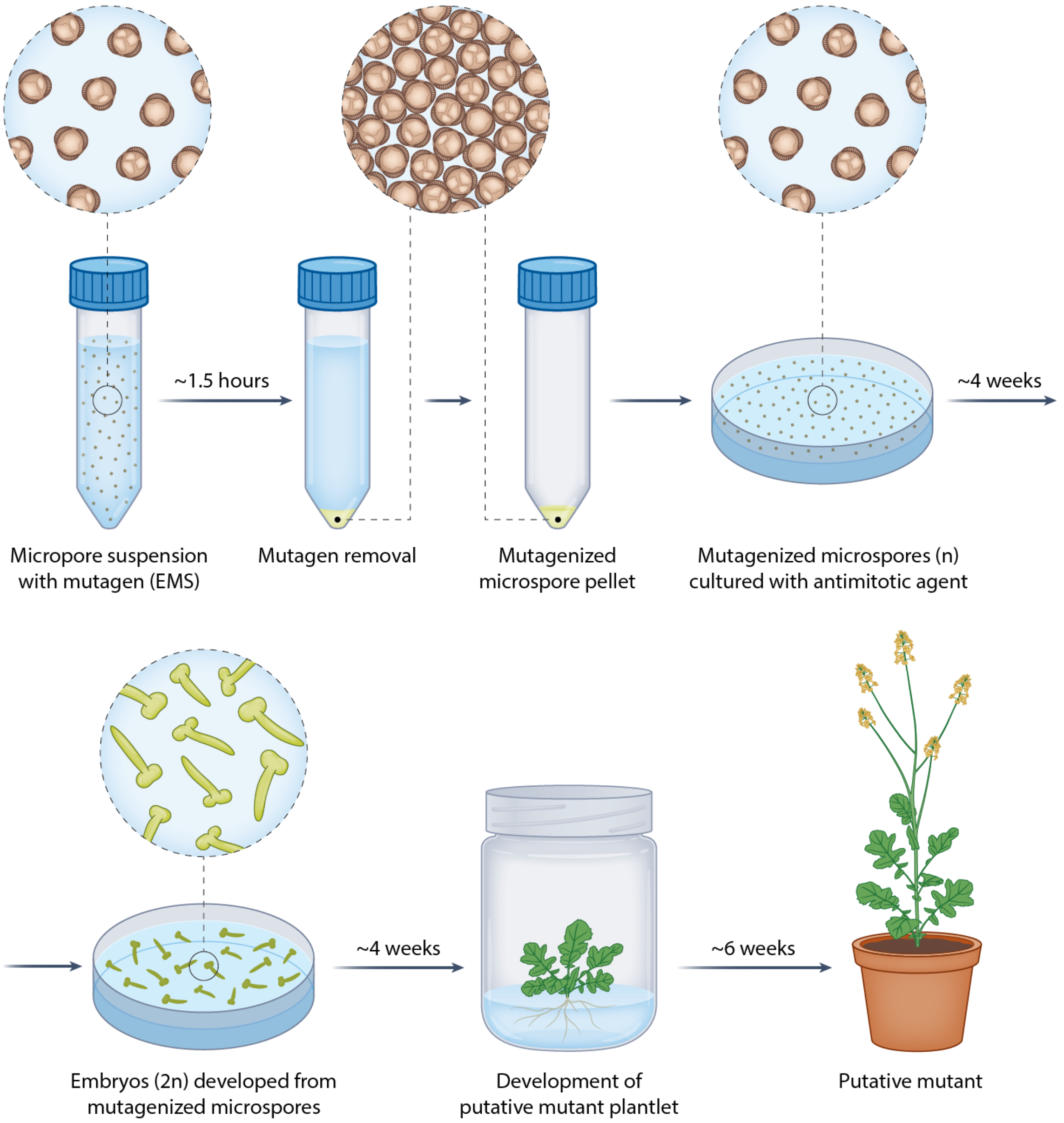

Supplementary Figure 1. Overview of the haploid microspore mutagenesis protocol.

Microspores of Brassica napus genotype NRCDH4079 were treated with varying concentrations of EMS (0–0.4%) and subsequently cultured to induce embryogenesis. The resulting embryos were regenerated into plantlets, leading to the development of haploid and doubled haploid (DH) mutant plants.

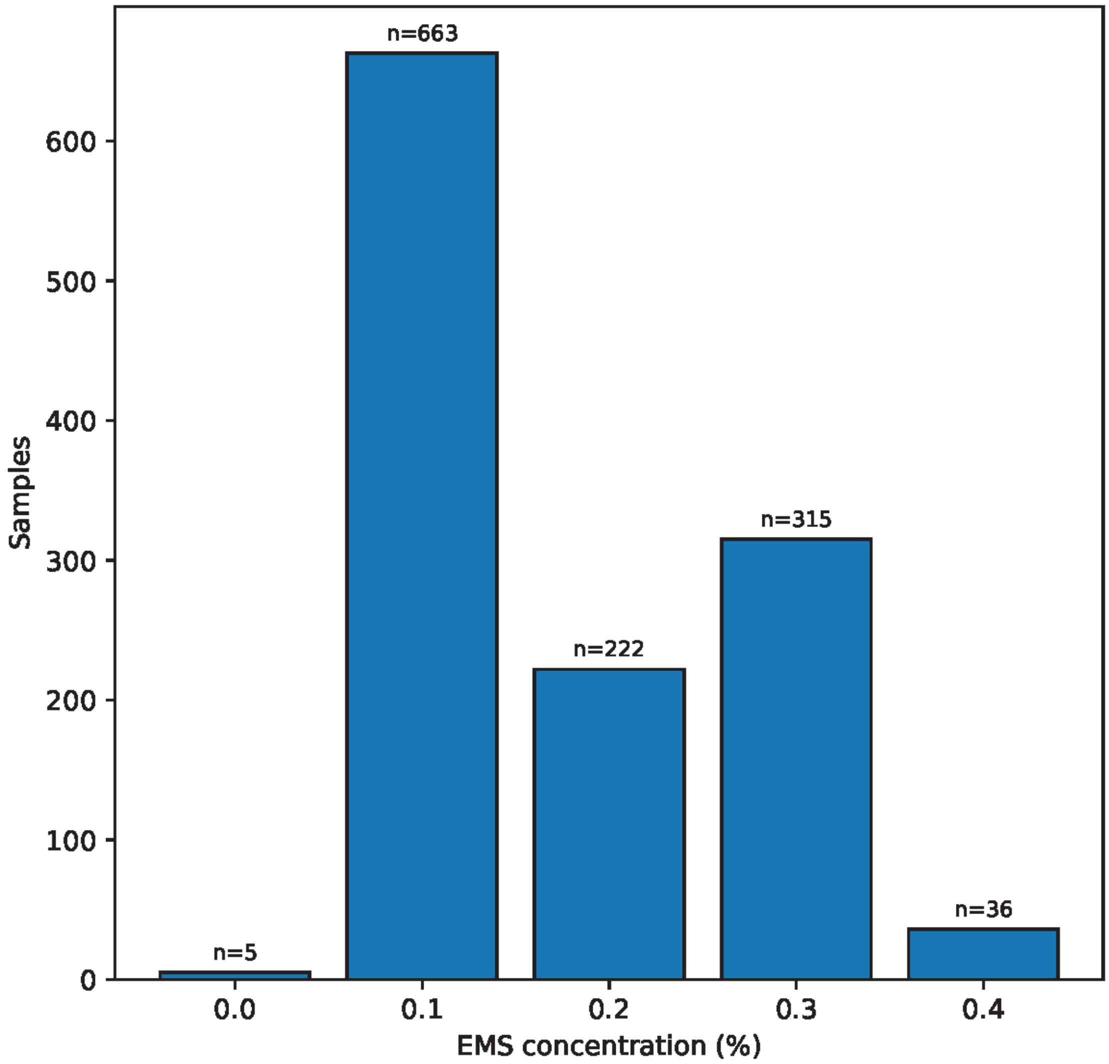

Supplementary Figure 2. Histogram showing the number of mutant lines recovered at each EMS concentration (0.0%, 0.1%, 0.2%, 0.3%, 0.4%).

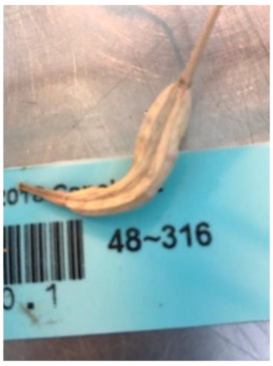

Fused pods

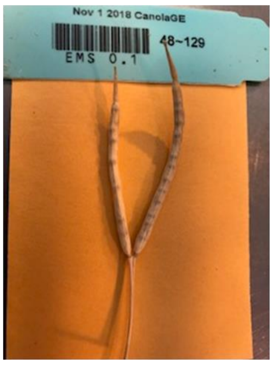

Two pods per pedicel

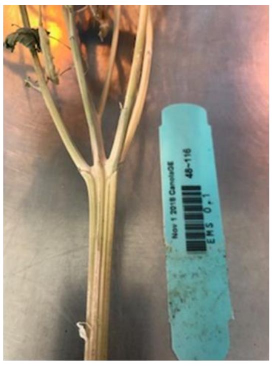

Fused branches/stem

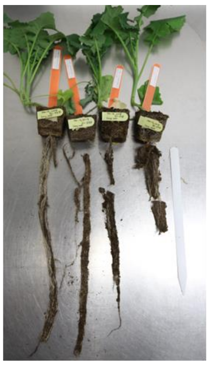

Variation in root volume

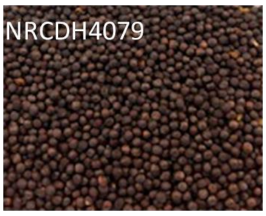

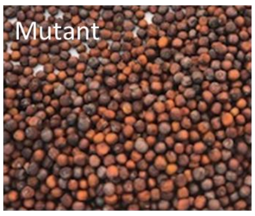

Seed color variation

Supplementary Figure 3. Phenotypes of interest observed in EMS mutagenized *B. napus* lines

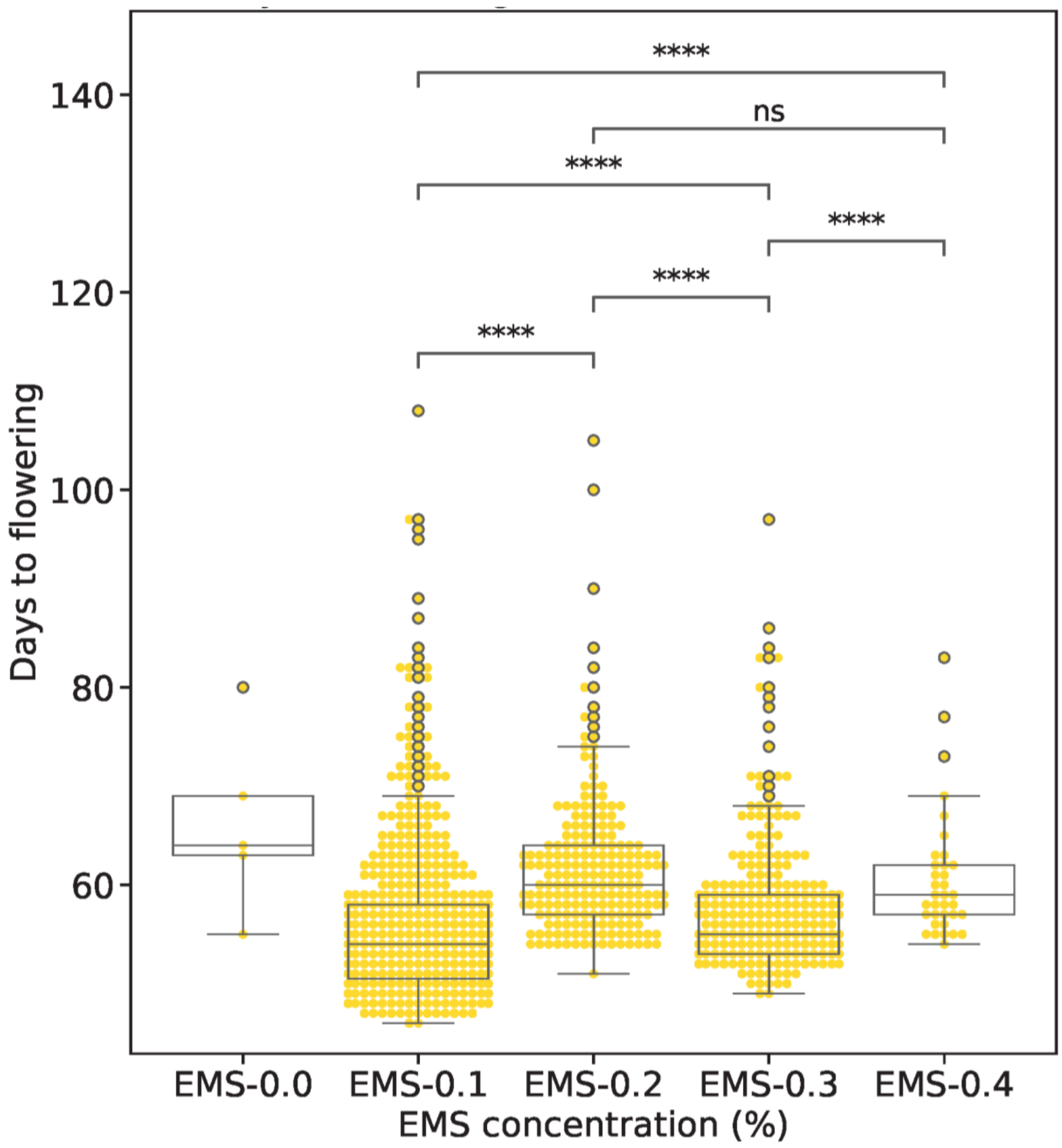

Supplementary Figure 4. Distribution of days to flowering phenotype across EMS concentrations in mutagenized population.

Box plots with overlaying swam plots showing days to flowering per sample for each sample separated out by EMS concentration. P-values are shown only for treated samples as there are only 5 samples in the control group. P-values are as follows: ns: 5.00e-02 < p <= 1.00e+00, *: 1.00e-02 < p <= 5.00e-02, **: 1.00e-03 < p <= 1.00e-02, ***: 1.00e-04 < p <= 1.00e-03, ****: p <= 1.00e-04.

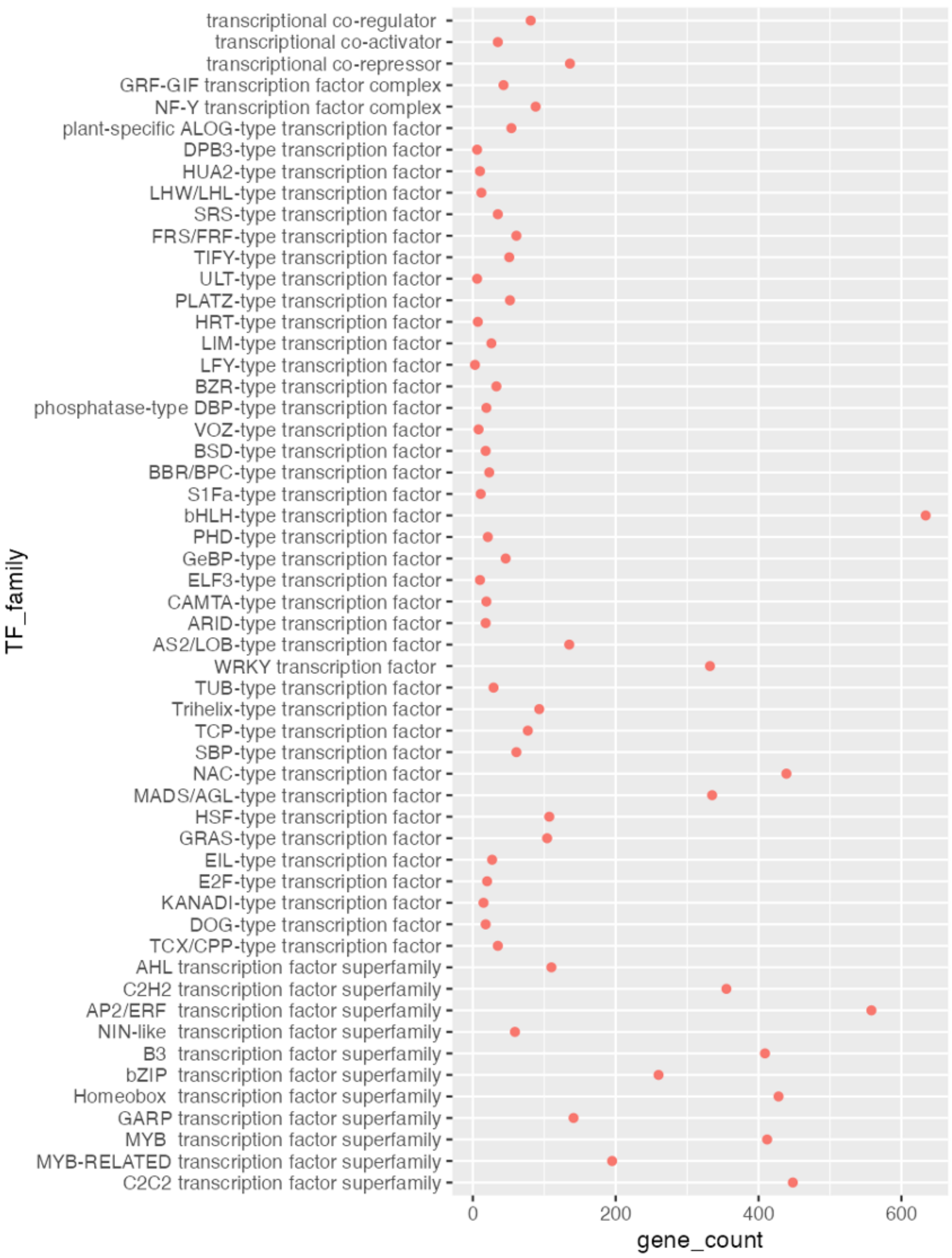

Supplementary Figure 5. Transcription factors (TF) families in the B. napus genotype DH4079 genome

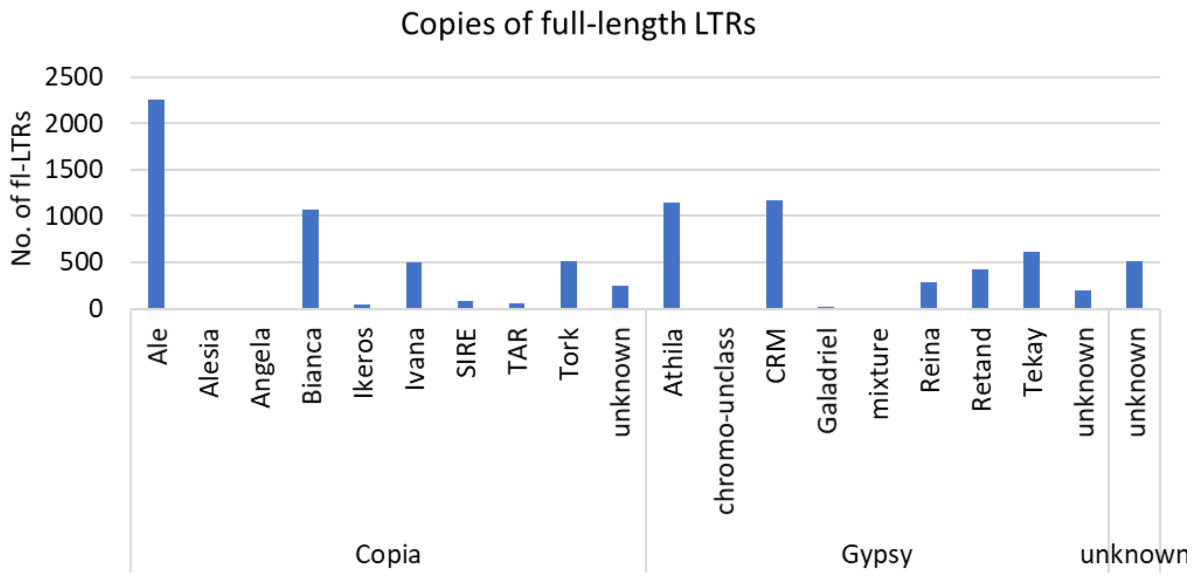

Supplementary Figure 6. Distribution of full-length LTRs in the B. napus genotype DH4079 genome

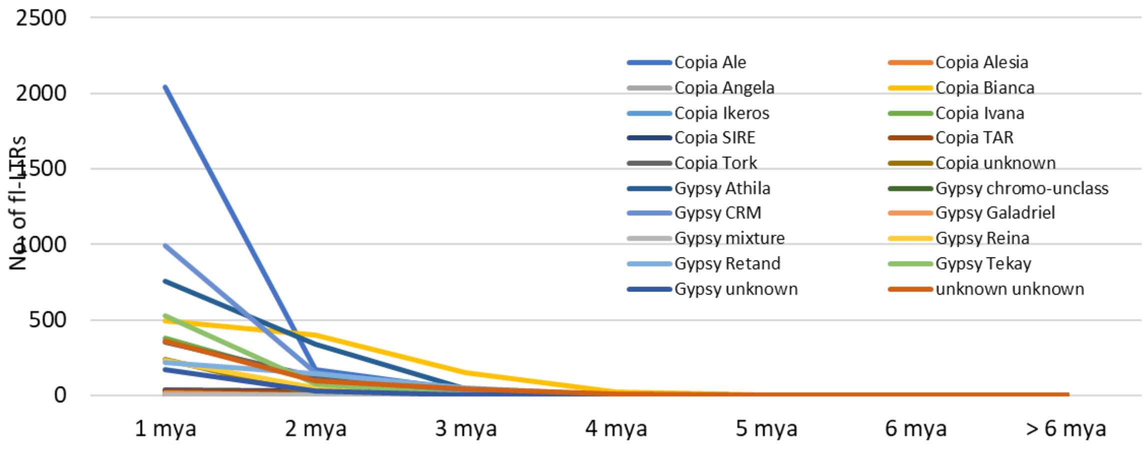

Supplementary Figure 7. Age distribution of full-length LTRs

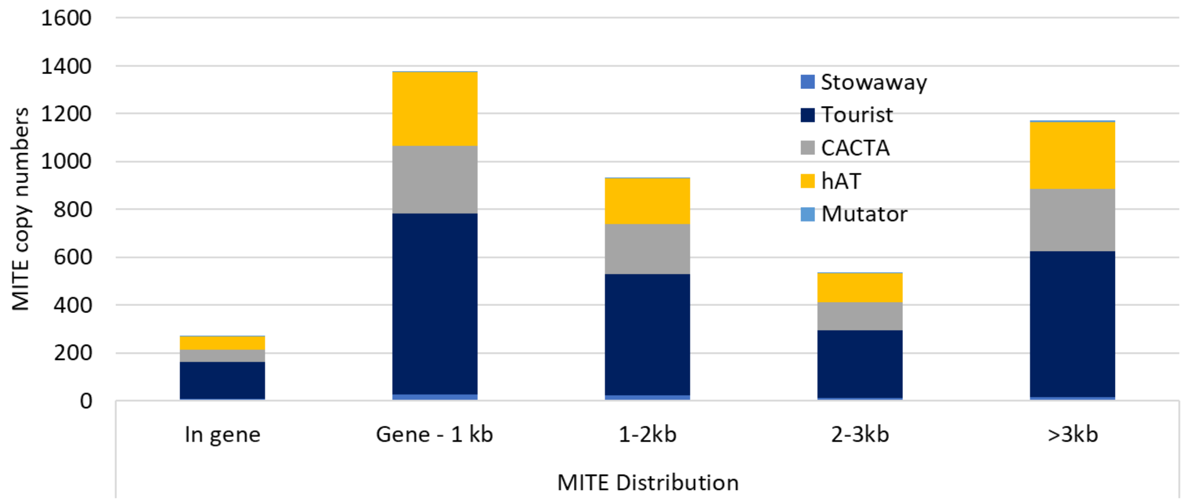

Supplementary Figure 8. Distribution of Miniature Inverted repeat transposable elements (MITEs)

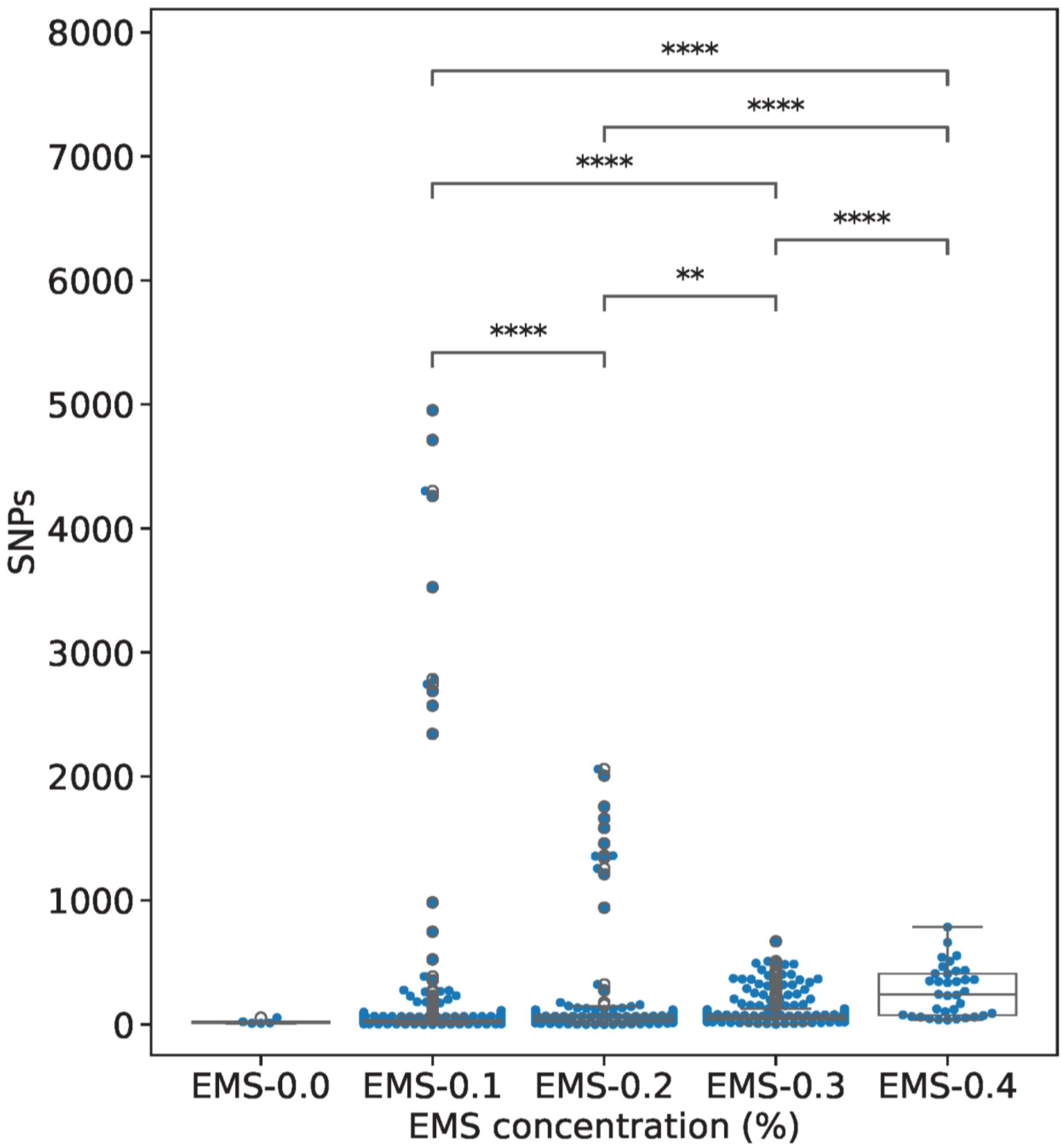

Supplementary Figure 9. Distribution of SNPs across EMS concentrations in mutagenized population

Box plots with overlaying swam plots showing the number of SNPs per sample for each sample separated out by EMS concentration. P-values are shown only for treated samples as there are only 5 samples in the control group. P-values are as follows: ns: 5.00e-02 < p <= 1.00e+00, *: 1.00e-02 < p <= 5.00e-02, **: 1.00e-03 < p <= 1.00e-02, ***: 1.00e-04 < p <= 1.00e-03, ****: p <= 1.00e-04.

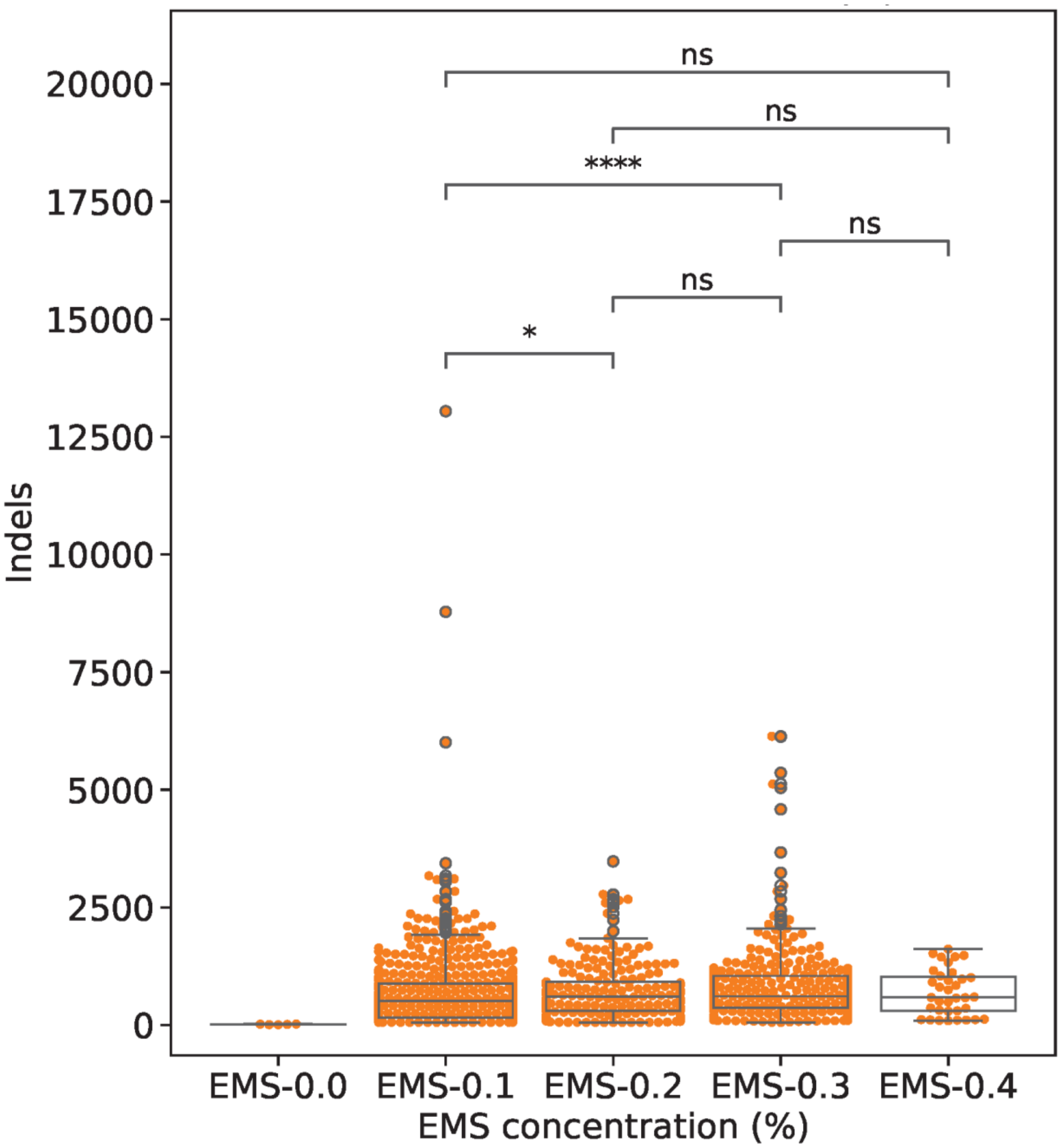

Supplementary Figure 10. Distribution of InDels across EMS concentrations in mutagenized population

Box plots with overlaying swam plots showing the number of SNPs per sample for each sample separated out by EMS concentration. P-values are shown only for treated samples as there are only 5 samples in the control group. P-values are as follows: ns: 5.00e-02 < p <= 1.00e+00, *: 1.00e-02 < p <= 5.00e-02, **: 1.00e-03 < p <= 1.00e-02, ***: 1.00e-04 < p <= 1.00e-03, ****: p <= 1.00e-04.

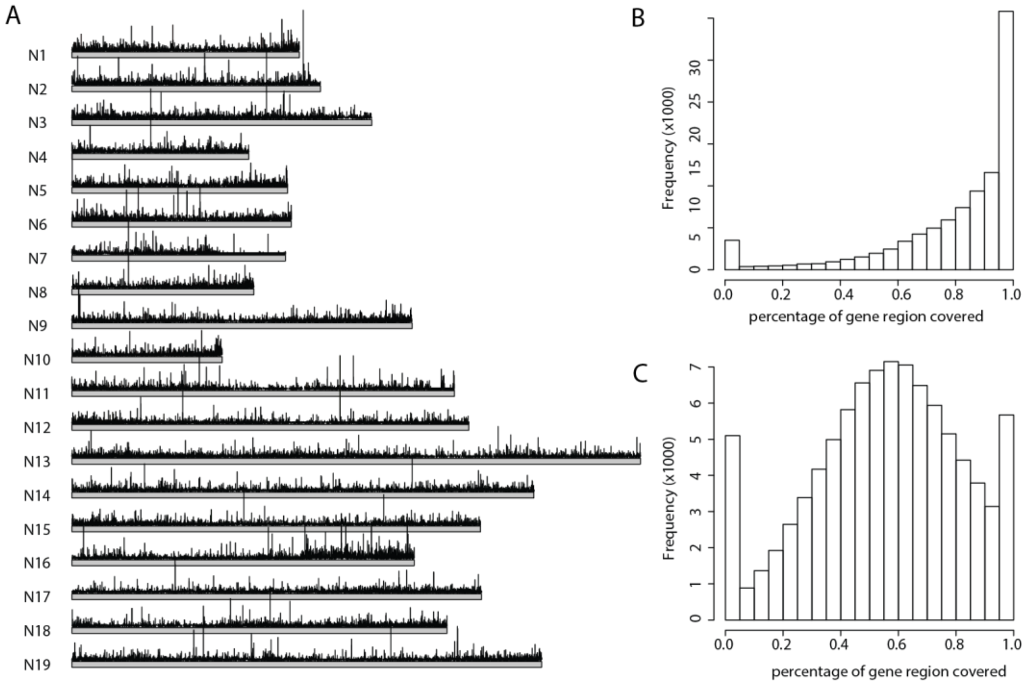

Supplementary Figure 11. Validation of the exome capture array.

A. Distribution of mapped reads per gene in a 16-plex assay, illustrating the efficiency and uniformity of read mapping across targeted genes.
B. Percentage of each gene region covered in the 16-plex assay, indicating the breadth of coverage achieved through the capture protocol.
C. Percentage of gene region covered in a 28-plex assay, allowing comparison of coverage efficiency between different multiplexing levels.
